# A transcriptomic signature mediated by HOXA9 promotes human glioblastoma initiation, aggressiveness and resistance to temozolomide

**DOI:** 10.18632/oncotarget.3150

**Published:** 2015-02-20

**Authors:** Marta Pojo, Céline S. Gonçalves, Ana Xavier-Magalhães, Ana Isabel Oliveira, Tiago Gonçalves, Sara Correia, Ana J. Rodrigues, Sandra Costa, Luísa Pinto, Afonso A. Pinto, José M. Lopes, Rui M. Reis, Miguel Rocha, Nuno Sousa, Bruno M. Costa

**Affiliations:** ^1^ Life and Health Sciences Research Institute (ICVS), School of Health Sciences, University of Minho, Campus de Gualtar 4710-057 Braga, Portugal; ^2^ ICVS/3B's - PT Government Associate Laboratory, Braga/Guimarães, Campus de Gualtar 4710-057 Braga, Portugal; ^3^ Centre of Biological Engineering/Department of Informatics, University of Minho, Campus de Gualtar 4710-057 Braga, Portugal; ^4^ Department of Neurosurgery, Hospital de Braga, Sete Fontes, 4710-243 São Victor, Braga, Portugal; ^5^ Department of Pathology, Hospital S. João, Alameda Professor Hernâni Monteiro, 4200-319 Porto, Portugal; ^6^ Institute of Molecular Pathology and Immunology at the University of Porto (IPATIMUP), Rua Dr. Roberto Frias s/n 4200-465 Porto, Portugal; ^7^ Medical Faculty, University of Porto, Alameda Professor Hernâni Monteiro, 4200-319 Porto, Portugal; ^8^ Barretos Cancer Hospital, Molecular Oncology Research Center, Rua Antenor Duarte Vilela, 1331 - Doutor Paulo Prata, Barretos - SP, 14780-000, Brasil

**Keywords:** Glioblastoma, prognosis, oncogene, temozolomide, *HOXA9*

## Abstract

Glioblastoma is the most malignant brain tumor, exhibiting remarkable resistance to treatment. Here we investigated the oncogenic potential of HOXA9 in gliomagenesis, the molecular and cellular mechanisms by which HOXA9 renders glioblastoma more aggressive, and how HOXA9 affects response to chemotherapy and survival. The prognostic value of *HOXA9* in glioblastoma patients was validated in two large datasets from TCGA and Rembrandt, where high *HOXA9* levels were associated with shorter survival. Transcriptomic analyses identified novel HOXA9-target genes with key roles in cancer-related processes, including cell proliferation, DNA repair, and stem cell maintenance. Functional studies with *HOXA9*-overexpressing and *HOXA9*-silenced glioblastoma cell models revealed that *HOXA9* promotes cell viability, stemness and invasion, and inhibits apoptosis. Additionally, *HOXA9* promoted the malignant transformation of human immortalized astrocytes in an orthotopic *in vivo* model, and caused tumor-associated death. *HOXA9* also mediated resistance to temozolomide treatment *in vitro* and *in vivo* via upregulation of BCL2. Importantly, the pharmacological inhibition of BCL2 with the BH3 mimetic ABT-737 reverted temozolomide resistance in HOXA9-positive cells. These data establish HOXA9 as a driver of glioma initiation, aggressiveness and resistance to therapy. In the future, the combination of BH3 mimetics with temozolomide should be further explored as an alternative treatment for glioblastoma.

## INTRODUCTION

Glioblastoma (GBM) is the most common and malignant glioma type of the central nervous system [1]. Annually ~10,000 new cases are diagnosed in the United States, and > 50,000 patients live with the disease [2]. The clinical evolution is poor and variable among patients [3], and ~32% of all diagnosed cases survive less than 1 year [2]. A pivotal phase III clinical trial in 2005 showed that temozolomide-based chemotherapy plus radiation was significantly more effective than radiation alone [4]. A more recent study validated the therapeutic benefit of radiotherapy with concomitant and adjuvant temozolomide as compared to radiotherapy alone [5]. The methylation status of the promoter region of *MGMT* is one of the most promising prognostic biomarkers of GBM: a methylated *MGMT* promoter is associated with a more effective tumor response to temozolomide and increased survival of GBM patients [6]. Mechanistically, *MGMT* methylation reduces gene expression, which decreases tumor cells' ability to repair temozolomide-induced DNA damages, thus increasing drug sensitivity [6]. However, this association is not universal, as some tumors with a methylated *MGMT* promoter do not benefit from temozolomide treatment, while others with unmethylated *MGMT* respond favorably [7]. Therefore, there is an emerging need in discovering new molecular markers of drug response.

An aberrant expression of many of the 39 *HOX* genes has been found in various human cancers, affecting several hallmarks of cancer, including increased proliferation, angiogenesis, invasion, and resistance to apoptosis [8–11]. In gliomas, several *HOX* genes were shown to be part of large gene expression signatures that are associated with the maintenance of GBM stem cells and therapy resistance [12–15]. Specifically, it was shown that *HOXA9* and *HOXA10* have prognostic value in adult and pediatric high-grade glioma patients [12, 13, 16]. While HOXA10 was recently shown to drive the expression of genes with critical roles in gliomagenesis [14] and to increase temozolomide resistance *in vitro* [15], the downstream mechanisms by which HOXA9 may contribute to poor outcomes in GBM patients have not been addressed. Given its prognostic value, a more complete understanding of the molecular targets and the functional consequences of HOXA9 activation on the establishment and maintenance of the malignant phenotype of glioblastoma is required.

In this report, we pinpoint the genome-wide transcriptome of HOXA9 in GBM and demonstrate its functional relevance in initiating gliomas *in vivo* using immortalized astrocytes and established GBM cells. We also present data showing that HOXA9 promotes several oncogenic features, including increased cell viability, invasion, and stem cell-like features, and decreased sensitivity to temozolomide treatment *in vitro* and *in vivo*, and identify BCL2 as a putative therapeutic target to improve the response of HOXA9-positive GBMs to temozolomide. Our findings provide new insights regarding strategies to target *HOXA9* overexpression in this incurable cancer.

## RESULTS

### *HOXA9* is overexpressed and has prognostic value in GBM patients

**Figure 1:**
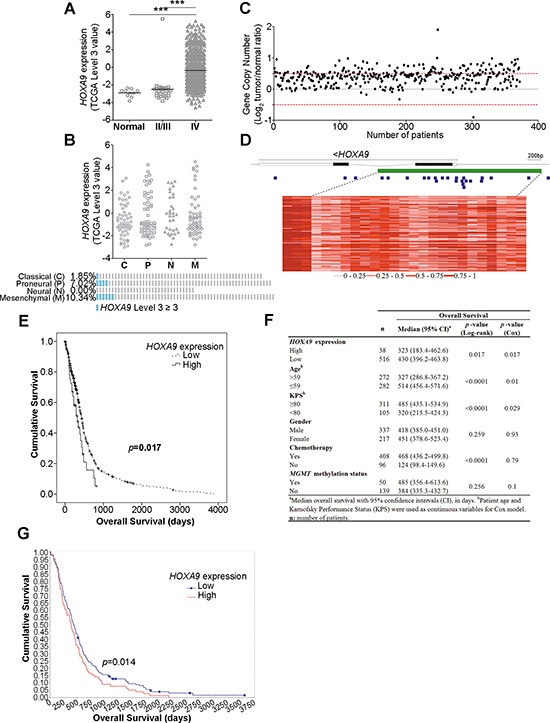
*HOXA9* expression is associated with WHO glioma grade and is an independent prognostic factor in glioblastoma patients **(A)** Expression levels of *HOXA9* in 10 unmatched normal controls, 27 lower-grade gliomas (LGG) and 572 glioblastoma (GBM) patients from TCGA. *HOXA9* is significantly overexpressed in GBM patients compared to LGG or normal samples (*** = *p* < 0.0001). **(B)** *HOXA9* high expression (TCGA level 3 ≥ 3) is more frequent in the mesenchymal (10.34%) and in the proneural subtypes (7.02%). **(C)** *HOXA9* gene copy number status in 372 GBM specimens from TCGA. *HOXA9* is amplified (Log_2_ Copy Number Tumor/Normal ≥ 0.5) in 31% (*n* = 114) of GBM samples. The normal copy number interval is between the red dashed lines. **(D)** Heatmap representation of DNA methylation levels (TCGA β-values) of the chromosomal region encompassing *HOXA9* in 74 GBM samples from TCGA. A total of 25 methylation probes (blue squares) were assessed, encompassing the CpG island (> 300 bp, represented in green). The color code (grades of red color corresponding to different methylation indexes) is shown below the heatmap. Each line corresponds to a patient and each column to a probe. The lines within *HOXA9* correspond to introns. **(E)** Kaplan-Meier survival curves of 554 GBM patients from TCGA indicate that patients whose tumors present high levels of *HOXA9* expression show a statistically significant shorter overall survival when compared to those whose tumors present lower levels of *HOXA9* (Log-rank test, *p*-value = 0.017). **(F)** Univariate and multivariate analyses of associations between *HOXA9* expression levels and survival of patients with glioblastoma (adjusted for patient age, KPS, gender, treatment with chemotherapy, and *MGMT* methylation status). **(G)** Kaplan-Meier survival curves of 181 GBM patients from REMBRANDT dataset confirms that patients whose tumors present high levels of *HOXA9* expression (78/181) present a statistically significant shorter overall survival when compared to those whose tumors present lower levels (Log-rank test, *p*-value = 0.014).

*HOXA9* expression was analyzed in WHO grades II/III glioma patients (27) and grade IV GBM patients (572) deposited in TCGA [17]. *HOXA9* was found to be highly overexpressed in a subset of GBM patients comparing to lower grades glioma (LGG, WHO grades II/III) patients and normal controls (Figure 1A), confirming that *HOXA9* is associated with glioma grade and may be important in tumor progression. According to the four GBM molecular subgroups [18], *HOXA9* overexpression was more frequent in the mesenchymal (10.34%) and in the proneural subtypes (7.02%; Figure 1B).

In order to explore putative mechanisms responsible for *HOXA9* overexpression in GBM, *HOXA9* copy number aberrations and DNA methylation levels were evaluated using the TCGA database (Figure 1C and 1D). *HOXA9* was found to be amplified in 31% (114/372) of GBM patients (Figure 1C), of which only 7.9% (9/114) presented high levels of *HOXA9* mRNA (*p* = 0.548, Chi-square test). Similarly, in the 25 methylation probes encompassing the *HOXA9* locus that were evaluated, most presented a consistent methylation pattern across all patients (Figure 1D). Together, these results suggest that gene amplification or methylation are not major drivers of *HOXA9* overexpression in glioma.

We have recently associated high levels of *HOXA9* expression with shorter overall survival (OS) of patients with GBM [16]. In order to validate this finding in a significantly larger and independent dataset, the prognostic value of *HOXA9* was evaluated in 554 GBM patients with available survival data from TCGA [17]. Patients whose tumors present high *HOXA9* expression had significantly shorter OS (median OS 323 days; 95% CI 183–463 days) than patients whose tumors express low *HOXA9* levels (median OS 430 days; 95% CI 396–464 days; Log-rank test *p*-value = 0.017; Figure 1E). Importantly, this association between *HOXA9* and OS was independent of other putative prognostic variables, including patient age, gender, Karnofsky performance status, *MGMT* methylation and treatment with chemotherapy, as indicated by multivariate analysis (Cox model *p*-value = 0.017; Figure 1F). Interestingly, in GBM patients presenting low levels of *MGMT* expression, *HOXA9* overexpression alone was sufficient to identify a subset of patients with shorter OS (median OS of 291 ± 75.4 and 435 ± 19.2 days in *HOXA9*-positive versus *HOXA9*-negative patients, respectively; Log-rank, *p*-value = 0.013). The prognostic value of *HOXA9* was additionally validated in an independent dataset from the REMBRANDT database (Log-rank, *p*-value = 0.014; Figure 1G).

Together, these data implicate *HOXA9* as a critical molecule in the malignant progression of gliomas, and validate its prognostic value in high-grade GBM patients.

### *HOXA9*-mediated transcriptomic signatures in GBM sustain cancer-related pathways

**Figure 2:**
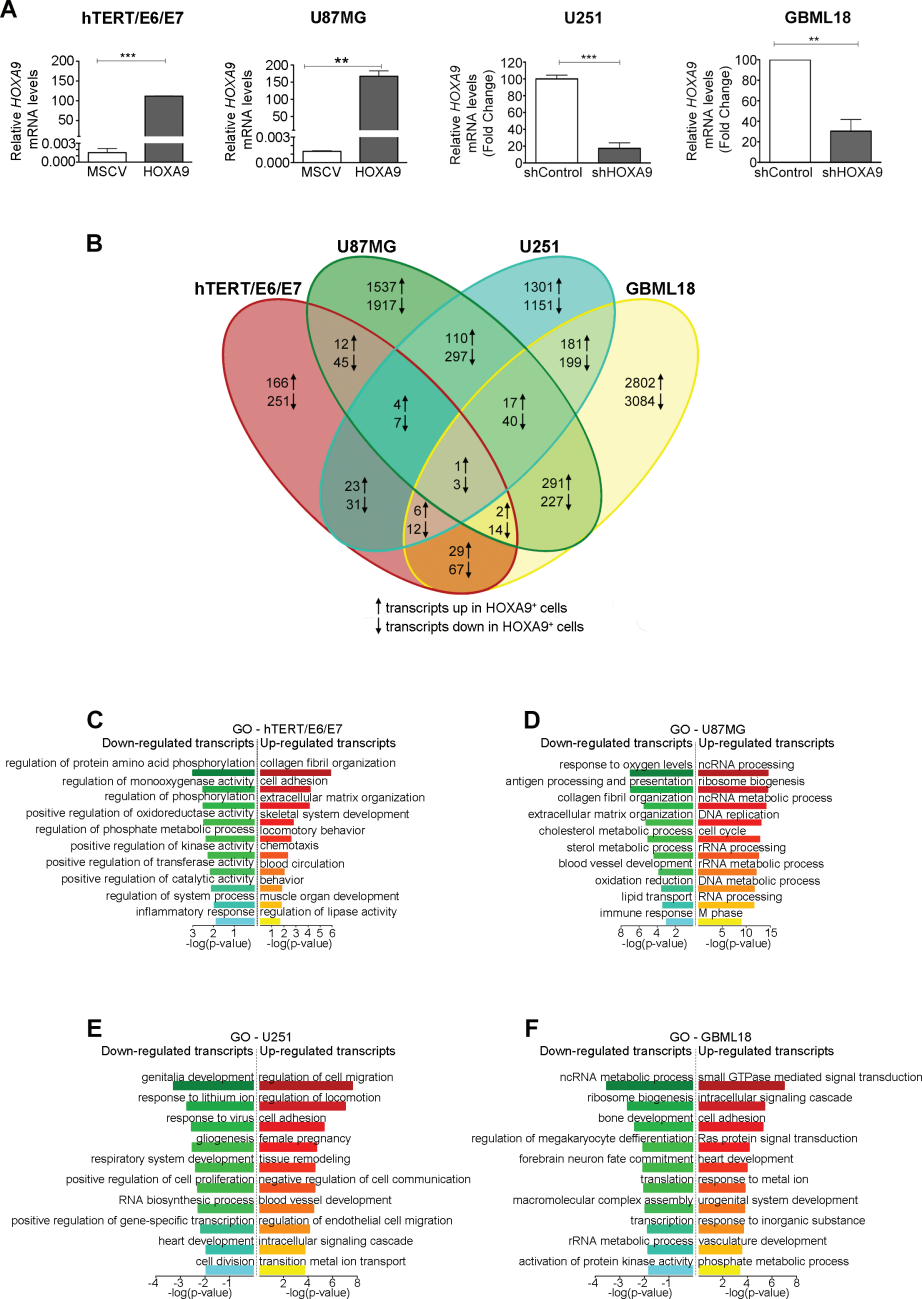
*HOXA9* transcriptomes in hTERT/E6/E7 human immortalized astrocytes, and in U87MG, U251 and GBML18 glioblastoma cell lines **(A)** qPCR confirming the overexpression of *HOXA9* in hTERT/E6/E7-HOXA9 and U87MG-HOXA9 cells and *HOXA9*-silencing in U251-shHOXA9 and GBML18-shHOXA9 cells, comparing to their respective control counterparts. **(B)** Venn diagram summarizing the number of differentially expressed transcripts in the microarray data in all cell lines. The numbers in each area represent the total number of transcripts within each intersection. **(C–F)** DAVID was used to query the HOXA9-transcriptome from each cell line (C, hTERT/E6/E7; D, U87MG; E, U251; F, GBML18), in order to identify enriched biological terms on the differentially expressed genes extracted from the microarray data. Statistically significant enriched GO terms are shown for each cell line.

The endogenous expression levels of *HOXA9* in a panel of glioma cell lines was evaluated by qPCR (Supplementary Figure 1). In order to provide the first characterization of HOXA9 targets on a genome-wide level in GBM, expression microarray analyses were performed in matched *HOXA9*-positive and *HOXA9*-negative human GBM cell models (U87MG-MSCV vs. U87MG-HOXA9, human immortalized astrocytes hTERT/E6/E7-MSCV vs. hTERT/E6/E7-HOXA9, U251-shControl vs. U251-shHOXA9, and a primary GBM cell line GBML18-shControl vs. GBML18-shHOXA9; Figure 2A and 2B). Due to *HOXA9* expression, 3454 probes were significantly differentially expressed in U87MG cells (1537 upregulated and 1917 downregulated), 417 probes in hTERT/E6/E7 cells (166 upregulated and 251 downregulated), 2452 probes in U251 cells (1301 upregulated and 1151 downregulated), and 5886 probes in GBML18 patient-derived primary cells (2802 upregulated and 3084 downregulated; Figure 2B and Supplementary Figures 4–9; GEO accession number GSE56517). Only a small subset of probes was consistently altered in the 3 GBM cell lines (17 probes upregulated and 40 downregulated due to HOXA9 expression in U87MG, U251 and GBML18 cells), which, as expected, was even smaller when integrating the non-tumor immortalized astrocytes (1 probe upregulated and 3 downregulated; Figure 2B), indicating that the transcriptome of HOXA9 is cell-type dependent, as previously suggested in leukemia models [19]. Gene-specific expression analyses in a subset of HOXA9 targets validated the array data (Supplementary Figure 2A–2D).

In order to understand the biological relevance of the HOXA9 transcriptome, functional clustering annotation and integration into Kyoto Encyclopedia of Genes and Genomes (KEGG), Reactome and Gene Ontology (GO) analyses were performed (Figure 2C–2F and Supplementary Figure 3A–3G). The HOXA9 transcriptome of non-tumoral hTERT/E6/E7 immortalized astrocytes revealed particular enriched pathways in KEGG (e.g. chemokine signaling pathway for HOXA9-upregulated genes, and cytokine-cytokine receptor interaction pathway for HOXA9-downregulated genes; Supplementary Figure 3A). These pathways have been found deregulated in the context of cancer, and associated with increased proliferation and invasion [20]. In the GO integration (Figure 2C), these cells showed significant enrichment mainly to biological processes related to cellular adhesion (HOXA9-upregulated genes) and regulation of protein activity (HOXA9-downregulated genes). No significant enrichments were observed for the Reactome pathways in these cells. In the 3 GBM cell lines, the HOXA9 transcriptomes were enriched for several cancer-related pathways in KEGG, GO and Reactome analyses, including genes involved in cell cycle, DNA replication and repair, RNA processing, cell adhesion and migration, vasculature development, and immune-related pathways (Figure 2D–2F and Supplementary Figure 3B–3G). Together, these data suggest the HOXA9-mediated genetic signatures in GBM cell models are associated with important cancer hallmarks that may favor tumor development, progression, and aggressiveness. Moreover, HOXA9-target genes may be important in sustaining the HOXA9-associated aggressive phenotype and poor prognosis observed in GBM patients.

**Figure 3:**
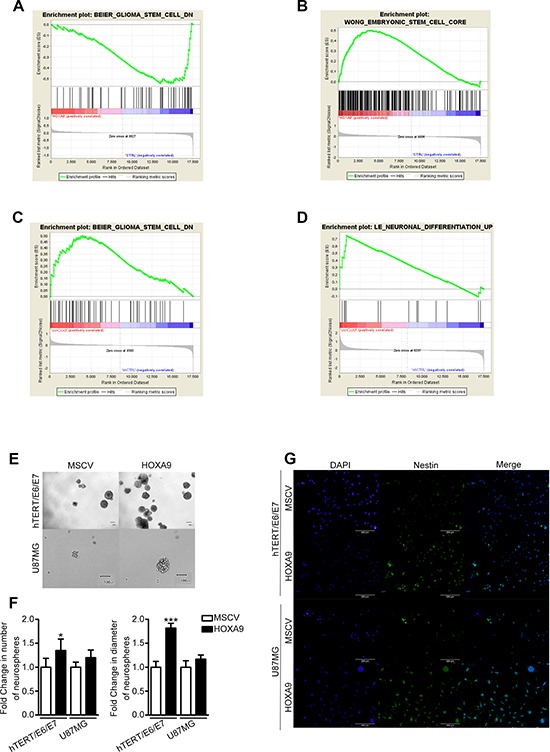
*HOXA9* transcriptomes are associated with cancer stem cell features **(A–D)** GSEA reveals that the HOXA9 transcriptomes in hTERT/E6/E7 **(A)** and U251 **(C)** cells are associated with transcriptional signatures of glioma stem-like cells (Enrichment Score, ES = −0.54, False Discovery Rate, FDR = 0.19; and ES = 0.50, FDR = 0.11, respectively); in U87MG cells **(B)**, the HOXA9 transcriptome is positively associated with genes that are upregulated in embryonic stem cells (ES = 0.50, FDR < 0.0001); in GBML18 cells **(D)**, HOXA9 transcriptome is inversely associated with genes upregulated during the neuronal differentiation (ES = 0.75, FDR = 0.21). **(E)** Representative phase contrast photographs of hTERT/E6/E7 and U87MG neurospheres are shown. **(F)** Quantification of neurospheres number and size for each cell line (*n* = 3; *p < 0.05; ***p < 0.001). **(G)** Immunofluorescence showing increased Nestin staining in HOXA9-positive cells.

We next explored the Connectivity Map tool to identify drugs that induce gene expression signatures similar to our HOXA9 transcriptome data. Interestingly, the top 30 gene expression signatures revealed associations with cancer cells treated with phosphatidylinositol 3-kinase (PI3K) pathway inhibitors, namely LY-294002, sirolimus, and wortmannin (Supplementary Figure 3H), suggesting that our HOXA9 transcriptomic signature in GBM cell models is modulated by these drugs. This is in accordance with our previous report showing that inhibition of the PI3K pathway in GBM cells inhibits *HOXA9* transcription [16], further supporting the validity of our microarray data. Other drug treatments that alter HOXA9-associated gene expression signature were found among the tested cell lines (Supplementary Figure 3H), including several anti-cancer drugs, particularly histone deacetylase (HDAC) inhibitors trichostatin A (TSA), vorinostat and MS-275 [21], which are able to promote cell cycle arrest and apoptosis [22], and tanespimycin and its analogues geldanamycin, alvespimycin, and monorden [23, 24], which are HSP90 inhibitors also currently tested as anti-cancer drugs. Together, our findings indicate that the HOXA9 transcriptome in GBM cells may be reverted by several drugs with potential anticancer effects, particularly those interfering with PI3K signaling, HDAC and HSP90 functions, which may be clinically valuable to revert HOXA9-driven transcriptomic signatures.

### *HOXA9* expression is associated with cancer stem cell features

HOXA9-associated transcriptional signatures queried with gene set enrichment analysis (GSEA) revealed that the HOXA9 transcriptome in hTERT/E6/E7 cells was inversely associated with genes downregulated in glioma stem cells (enrichment score, ES = −0.54, false discovery rate, FDR = 0.19; Figure 3A), leukemia stem-like cells, and hematopoietic stem cells (ES = −0.44, FDR = 0.21; ES = −0.45, FDR = 0.20, respectively; Supplementary Figure 10A and Supplementary Table 1). Concordantly, HOXA9 target genes in U87MG cells were significantly associated with genes upregulated in embryonic stem cells (ES = 0.50, FDR < 0.0001; Figure 3B), DNA repair, and cycling gene expression signatures (ES = 0.54, FDR < 0.0001; and ES = 0.38, FDR = 0.007, respectively; Supplementary Figure 10B and Supplementary Table 1). Similarly, the transcriptome of HOXA9-silenced U251 cells is significantly associated with genes depleted in glioma stem cells and normal (embryonic, neural and hematopoietic) stem cells (ES = 0.50, FDR = 0.11 and ES = 0.43, FDR = 0.22, respectively; Figure 3C, Supplementary Figure 10C and Supplementary Table 1); in GBML18 cell line, HOXA9-downregulation enriched for genes upregulated during neuronal differentiation and in quiescent chronic myeloid leukemia cells (ES = 0.75, FD*R* = 0.21 and E*S* = 0.70, FD*R* = 0.21, respectively; Figure 3D, Supplementary Figure 10D and Supplementary Table 1). Together, these analyses suggest that the HOXA9-transcriptome is enriched for genes involved in stem-like cell features, a critical hallmark of cancer. To test this hypothesis, we evaluated the capacity of these cells to form neurospheres *in vitro*, which has been associated with stemness potential [25]. *HOXA9* significantly increased the size and number of neurospheres in hTERT/E6/E7 cells as compared to their HOXA9-negative counterparts, which was also observed in a lower extent in U87MG cells (Figure 3E and 3F). Additionally, the expression of the neural stem cell marker Nestin was significantly higher in both hTERT/E6/E7 and U87MG *HOXA9* positive cells (Figure 3G). Together, these data suggest *HOXA9* increases the stemness capacity of these cells.

### *HOXA9* expression is associated with tumor aggressiveness in GBM xenograft models

**Figure 4:**
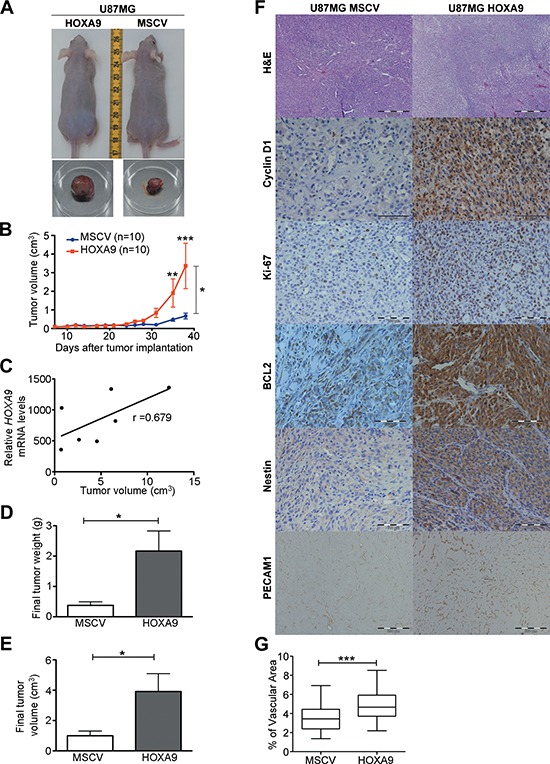
*HOXA9* accelerates tumor growth *in vivo* **(A)** U87MG cells were subcutaneously xenografted into nude mice; representative photographs of *in vivo* and *ex vivo* tumors are shown. **(B)** Longitudinal assessment of tumor volumes showing increased growth rates of U87MG-HOXA9 tumors as compared to *HOXA9*-negative tumors. **(C)** Positive correlation between expression levels of *HOXA9* and tumor volume. **(D)** and **(E)** Final tumor volumes (D) and weights (E) are significantly higher in *HOXA9*-positive tumors (*n* = 10). **(F)** Hematoxilin-eosin and immunohistochemical staining showing that *HOXA9*-positive tumors present higher protein levels of Cyclin D1, Ki-67, BCL2, Nestin, and PECAM1. **(G)** Quantification of the % of vascular area based on PECAM1 staining, showing increased angiogenic potential of U87MG-HOXA9 tumors. Statistical differences were calculated by two-way ANOVA (B) and Spearman correlation (C), or *t*-tests (D and E) (*p < 0.05; **p < 0.01; ***p < 0.001).

Considering that stem cell properties have been linked with tumor malignancy [14, 26, 27], together with our microarray data implicating the HOXA9 transcriptome in typical hallmarks of cancer (Supplementary Figure 3, Supplementary Figure 10 and Supplementary Table 1), we hypothesized that HOXA9 could play a role in tumor growth *in vivo*. To test this hypothesis, we established subcutaneous GBM xenograft models with U87MG cells. Tumors derived from U87MG-HOXA9 cells presented increased growth rates and were significantly larger than U87MG-MSCV cells (Figure 4A–4E). Interestingly, the final tumor volume was significantly correlated with *HOXA9* expression levels (Figure 4C), further strengthening the relevance of *HOXA9* on tumor growth kinetics *in vivo*. Histologically, *HOXA9* positive tumors presented a higher proliferative activity, as indicated by increased Ki-67 staining, which includes mostly neoplastic cells but also non-neoplastic cells (e.g. endothelial and stromal cells; Figure 4F). A higher expression of Nestin was also observed, supporting an increased stemness potential of *HOXA9*-positive cells *in vivo*. Furthermore, the expression levels of Cyclin D1 and BCL2 were also increased in U87MG-HOXA9 tumors *in vivo* (Figure 4F). These proteins are critical regulators of cell cycle and cell death, respectively, and were identified as HOXA9 targets in the microarray data. Tumor-associated angiogenesis, another critical hallmark of GBM, as assessed by PECAM1 protein staining, was significantly increased in *HOXA9*-positive tumors (Figure 4F and 4G). Together, these data establish *HOXA9* as a critical mediator of GBM growth and aggressiveness *in vivo*, and identifies critical molecular mediators sustaining this malignant phenotype.

### *HOXA9* expression contributes to glioma initiation and causes glioma-associated death

**Figure 5:**
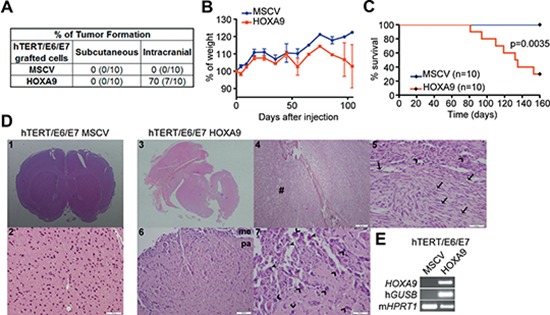
*HOXA9* induces glioma initiation and tumor-associated death in intracranial orthotopic xenografts **(A)** Nude mice were injected with *HOXA9*-negative and *HOXA9*-positive hTERT/E6/E7 immortalized astrocytes, either subcutaneously or intracranially. No tumor formation was observed in the subcutaneous model, regardless of *HOXA9* expression. In the intracranial orthotopic model, *HOXA9*-positive hTERT/E6/E7 cells originated tumors in the majority of tested mice (70%). **(B)** Mice intracranially injected with hTERT/E6/E7-HOXA9 cells display glioma-related loss of weight. **(C)** Kaplan-Meier survival curves showing tumor-associated death exclusively in mice bearing *HOXA9*-positive cells (Log-rank test, *p* = 0.0035). **(D)** Histological characterization of mice brains orthotopically-injected with hTERT/E6/E7-control or hTERT/E6/E7-HOXA9 cells. Brains from animals injected with *HOXA9*-negative cells display a normal, non-malignant appearance (images 1 and 2 at 20x and 200x magnification, respectively), while *HOXA9*-positive cells formed tumors displaying characteristic hallmarks of malignant gliomas (images 3, 4, 5, 6 and 7 at the magnification of 20x, 40x, 200x, 100x and 200x, respectively), including pleomorphic and spindle shape tumor cells (arrows), high mitotic activity (open arrowheads), prominent nuclear polymorphism (closed arrowheads), and infiltration to meninges and brain parenchyma (6 and 7). In image 4, cells infiltrating bone and soft tissues (#) presented a more sarcomatoid appearance, amplified in image 5. **(E)** RT-PCR analysis confirmed the expression of human *GUSB* housekeeping gene and *HOXA9* exclusively in *HOXA9*-positive xenografts. Me = meninges; pa = brain parenchyma.

Given our data implicating HOXA9 as a key molecule in mediating several aspects of cancer aggressiveness and stem cell characteristics, we hypothesized that HOXA9 may also play an important role in the tumorigenic process, possibly facilitating carcinogenesis initiation. To test this hypothesis, we evaluated the influence of HOXA9 on the capacity of hTERT/E6/E7 cells, a non-tumorigenic cell line [28], to establish tumors *in vivo*. In a subcutaneous model, hTERT/E6/E7 cells did not form tumors, regardless of *HOXA9* expression status (Figure 5A). Considering the relevance of the interplay between tumor cells and its microenvironment, we investigated the tumorigenic potential of these cells in an orthotopic intracranial model. Strikingly, while *HOXA9*-negative hTERT/E6/E7 cells did not form tumors, the expression of *HOXA9* in hTERT/E6/E7 rendered these cells highly tumorigenic in the brain, accompanied by glioma-related symptomatology and death (Figure 5A–5E). Histological analyses of the brains revealed that hTERT/E6/E7-HOXA9 tumors displayed hallmark features of malignant gliomas, including pleomorphic and spindle shape tumor cells, with prominent nuclear polymorphism and mitotic activity, and were highly infiltrative throughout the meninges and brain parenchyma. In some cases, tumors grew outside the brain, infiltrating bone and soft tissues, where cells displayed a more sarcomatoid appearance (Figure 5D). These data clearly establish HOXA9 as a novel critical molecular driver in the initial steps of malignant transformation of brain gliomas.

### *HOXA9* increases GBM aggressiveness by affecting cell viability, death, invasion and resistance to temozolomide *in vitro*

**Figure 6:**
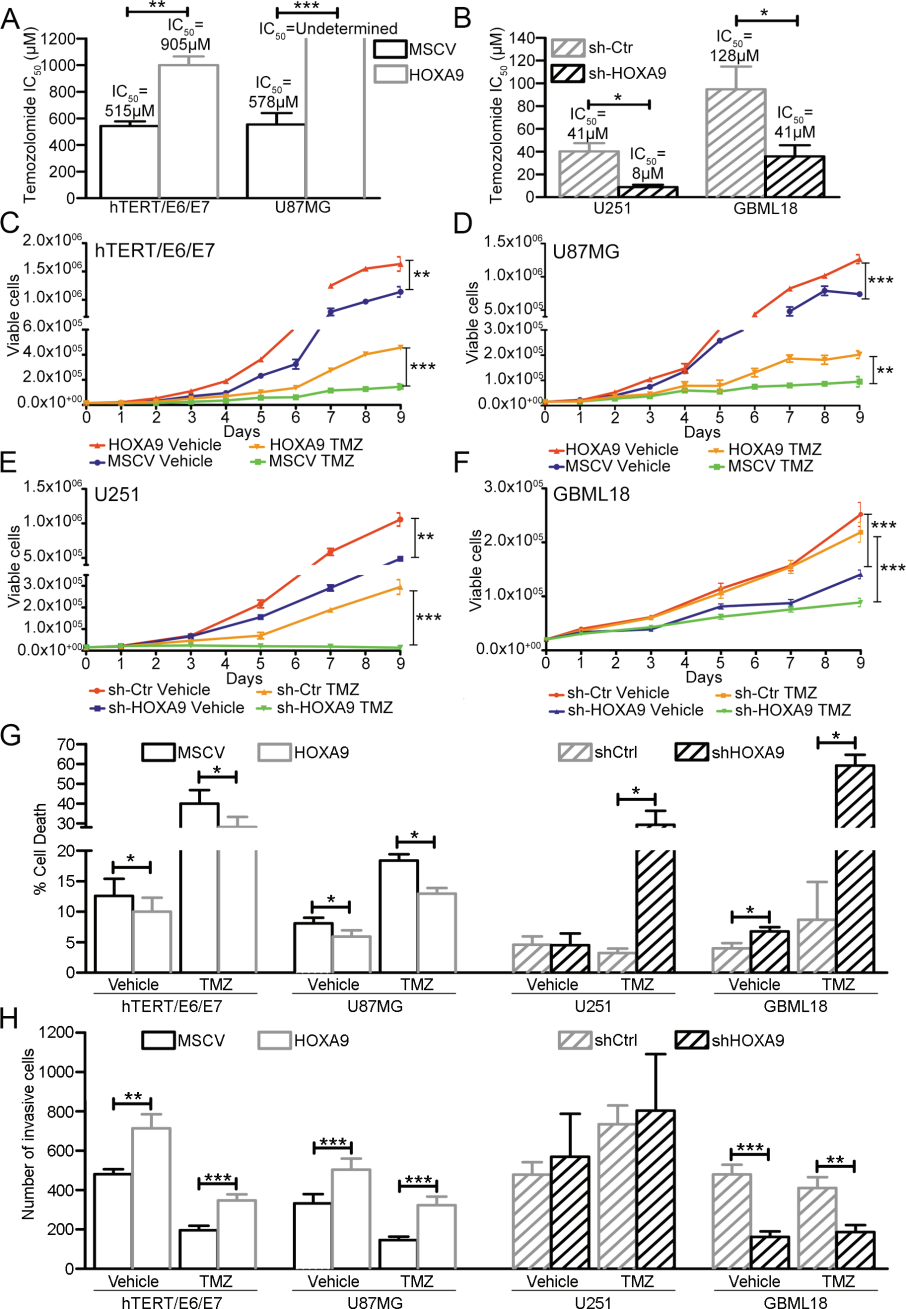
Functional roles of HOXA9 in GBM cell viability, death, and invasion, under basal conditions and temozolomide treatment **(A** and **B)** Determination of the half inhibitory concentration (IC_50_) values after 6 days of temozolomide (TMZ) treatment in *HOXA9*-positive or *HOXA9*-negative hTERT/E6/E7 and U87MG (A), and *HOXA9*-silenced or control U251 and GBML18 (B) cell lines. **(C–F)** Cell viability trypan blue assays in *HOXA9*-negative/low or *HOXA9*–positive/high hTERT/E6/E7 (C), U87MG (D), U251 (E) and GBML18 (F) cells, exposed to temozolomide or vehicle. **(G)** Cell death was evaluated by annexin V staining followed by flow cytometry in *HOXA9*-positive/high and *HOXA9*-negative/low hTERT/E6/E7, U87MG, U251 and GBML18 cell lines, both in basal conditions and after exposure to temozolomide (TMZ). *HOXA9* expression decreases cell death of all GBM cell models, both in basal conditions and after TMZ treatment, except in basal conditions for U251 cell line. **(H)** Cell invasion in the same cell lines, both in basal conditions and after exposure to TMZ. *HOXA9* increases the invasion of hTERT/E6/E7, U87MG, and GBML18 cells. U251 cells did not show significant differences in invasion profiles due to *HOXA9* levels or TMZ treatment. Results are representative of at least three independent experiments, performed in triplicates (data points represent mean ± SEM). Statistical differences were calculated by Student's *t*-test (panels A, B, G, H) and two-way ANOVA (panels C–F) (*p < 0.05; **p < 0.01; ***p < 0.001).

Our data implicated HOXA9 in several hallmarks of cancer, including cell proliferation, invasion, DNA repair pathways, and cancer stem cell features (Figure 3, Supplementary Figure 3 and Supplementary Figure 10 and Supplementary Table 1). Since most of these characteristics have been implicated in resistance to treatments, we performed an integrated set of functional assays to investigate HOXA9's effects on these processes, both in basal conditions and under temozolomide-treatment, the gold standard chemotherapeutic agent for GBM patients. Both hTERT/E6/E7 and U87MG cells overexpressing *HOXA9* presented significantly higher temozolomide IC_50_ values than their respective control cell lines (Figure 6A). In agreement with the overexpression models, we observed a decrease in temozolomide IC_50_ values for both cell lines after *HOXA9* downregulation (Figure 6B). Time-course viability assays were further evaluated by trypan blue exclusion (Figure 6C–6F) and MTS reduction (Supplementary Figure 11A–11D). Globally, both assays revealed that all *HOXA9*-high cell lines hTERT/E6/E7, U87MG, U251, and GBML18 (red lines) presented higher cell viability in basal conditions than their respective *HOXA9*-low counterparts (blue lines; Figure 6C–6F and Supplementary Figure 11A–11D), and that temozolomide-mediated cytotoxicity was significantly more pronounced in *HOXA9*-low cells than in *HOXA9*-high cells (Figure 6C–6F and Supplementary Figure 11A–11D), collectively indicating that *HOXA9* contributes to temozolomide resistance in GBM cells. We next assessed the influence of *HOXA9* expression in cell death by annexin V staining followed by flow cytometry. In basal conditions, most *HOXA9*-high cells presented significantly lower levels of cell death than their respective *HOXA9*-low counterparts (Figure 6G). In addition, all *HOXA9*-high cells presented significantly lower levels of temozolomide-mediated cell death than *HOXA9*-low cells (Figure 6G). An important feature of GBM is its striking ability to invade adjacent brain tissue, which is a major cause of tumor recurrence and resistance to therapy. Using invasion assays, we observed that *HOXA9* significantly increases the invasive capacity of hTERT/E6/E7, U87MG, and GBML18 cells, both in basal conditions and after treatment with temozolomide. In contrast, no significant changes in invasion were observed in U251 cells (Figure 6H). Since invasion and migration are intimately-related cellular processes, we also evaluated the migration capacity of U251 cells by wound healing assays (Supplementary Figure 12). Interestingly, U251-shControl cells presented significantly higher migration rates than U251-shHOXA9 counterparts. Together, our data consistently establishes the functional relevance of *HOXA9* expression in several hallmarks of GBM cells behavior, by affecting key cellular processes such as cell viability, death, invasion, and drug resistance, which together may dictate the more aggressive behavior and poorer clinical outcome of HOXA9-positive GBMs.

### *HOXA9* expression associates with shorter survival and increased resistance to temozolomide in orthotopic GBM xenograft models

**Figure 7:**
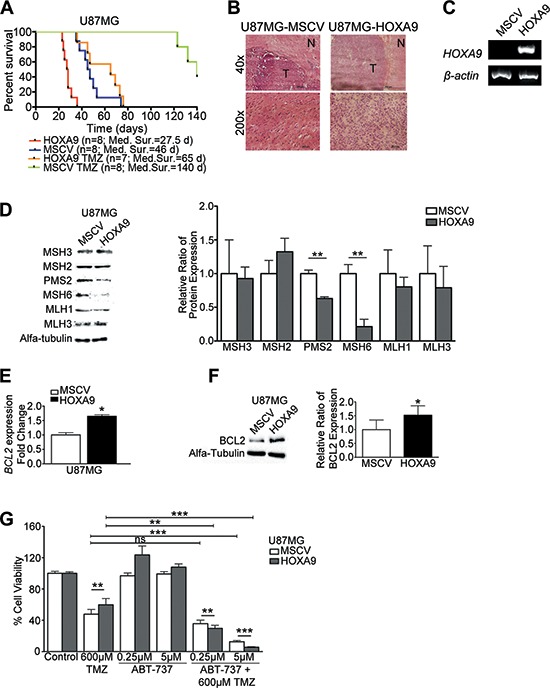
HOXA9 decreases overall survival and increases resistance to temozolomide *in vivo* via mismatch-repair and BCL2 proteins **(A)** Kaplan-Meier survival curves for *in vivo* orthotopic intracranial GBM models. While temozolomide (TMZ) treatment successfully increased overall survival, *HOXA9*-positive tumors cause significantly decreased overall survival in both untreated and TMZ-treated animals (Log-rank test HOXA9 vs MSCV, *p* = 0.0001; HOXA9 TMZ vs MSCV TMZ, *p* < 0.0001). **(B)** Hematoxylin-eosin staining of mice brains showing a well-delimited tumor area (*T * ) with GBM-like features, and surrounding non-tumor brain tissue (*N*). **(C)** RT-PCR analysis confirmed the expression of *HOXA9* in brain tumors derived from U87MG-HOXA9-xenografted cells. **(D)** Western Blot to mismatch-repair proteins (MMR), showing that *HOXA9* decreases the expression of PMS2 and MSH6 proteins in U87MG cells *in vitro*. **(E)** and **(F)** qPCR and Western blot analyses of BCL2 mRNA and protein levels, respectively, showing *HOXA9* significantly increases BCL2 levels. Results are representative of three independent experiments for each blot. **(G)** Cell viability of U87MG *HOXA9*-negative or -positive cells after 4 days of treatment with vehicle, Temozolomide (TMZ), ABT-737 (BCL2 inhibitor), or both. *HOXA9*-positive cells are significantly more sensitive to the combination of TMZ and ABT-737 than *HOXA9*-negative cells. Results are representative of three independent experiments, performed in triplicates (data points represent mean ± standard deviation). Statistical differences calculated by Student *t*-tests (*p < 0.05; **p < 0.01; ***p < 0.001).

In order to validate the prognostic value of *HOXA9* observed in GBM patients (Figure 1E–1G, and [16]), and its relevance in temozolomide chemo-resistance observed *in vitro* (Figure 6 and Supplementary Figure 11), we established intracranial orthotopic GBM xenografts with U87MG-MSCV and U87MG-HOXA9 cells in nude mice (Figure 7A). Untreated animals bearing U87MG-HOXA9-derived tumors presented significantly lower overall survival (median 28 days) than their respective negative counterparts (median 46 days; Log rank test, *p* = 0.0001; Figure 7A). Additionally, while treatment with temozolomide was able to significantly extend overall survival of all animals (median OS increased from 46 to 140 days in mice bearing U87MG-MSCV tumors, *p* < 0.0001; and median OS from 28 to 65 days in mice bearing U87MG-HOXA9 tumors, *p* = 0.0002; Figure 7A), those bearing *HOXA9*-expressing tumors presented a significantly shorter overall survival than animals bearing *HOXA9*-negative tumors (*p* < 0.0001; Figure 7A). Histological and RT-PCR analyses confirmed tumor formation and *HOXA9* expression levels in tumors excised from mice brains (Figure 7B and 7C, respectively). Taken together, our *in vivo* experiments specifically establish HOXA9 as a solid biomarker of prognosis in GBM and its relevance in determining temozolomide chemo-resistance.

### *HOXA9* affects important pathways involved in temozolomide response

The most well established mechanisms of temozolomide resistance include MGMT, base-excision repair (BER), and mismatch-repair (MMR) proteins [29]. In order to understand the mechanisms by which HOXA9 may affect drug resistance and mice survival, we investigated if HOXA9 affects these key pathways. By using methylation-specific PCR, we observed that U87MG cells presented a methylated *MGMT* promoter, independently of *HOXA9* expression (Supplementary Figure 13A). Concordantly, no differences in *MGMT* mRNA expression and protein levels were detected (Supplementary Figure 13B and 13C). Thus, the HOXA9-mediated temozolomide resistance in U87MG cells is not likely mediated by MGMT. Similarly, the levels of BER proteins (PCNA, PARP1, APEX1, and XRCC1) were not significantly affected by *HOXA9* (Supplementary Figure 13D). In contrast, the expression of some key proteins of the MMR pathway, including PMS2 and MSH6, was significantly decreased due to *HOXA9* (Figure 7D). Since a functional MMR pathway is crucial for temozolomide sensitivity by inducing apoptosis after DNA damage, the down-regulation of these key MMR proteins in HOXA9-positive U87MG cells may be an additional mechanism of temozolomide resistance.

Critically, it is not straightforward to therapeutically intervene at proteins of the MMR pathway in the clinical setting. Thus, we revisited our microarray data to identify other putative mechanisms of drug resistance. Interestingly, we identified several genes involved in apoptosis and drug response that were differentially expressed in U87MG-HOXA9 cells (e.g., upregulation of anti-apoptotic *BCL2*, *TRAIP*, *BAG1*, *BAG5*, *API5*, *SET*; and downregulation of pro-apoptotic *NLRP1*, *BLID*, *APLP1*, *ADCK3*). Of these, *BCL2* was particularly interesting because it was one of the top 50 most significantly up-regulated genes in our HOXA9 array data (Supplementary Figure 5). BCL2 is a critical anti-apoptotic protein involved in resistance to DNA damaging agents in many cancer types [30], for which pharmacological inhibitors are available, some of which have shown promising results in the treatment of solid tumors [31, 32]. Thus, we investigated how *HOXA9* may affect temozolomide response via BCL2. We first confirmed that BCL2 is significantly overexpressed at the mRNA and protein levels in HOXA9-positive cells (Figure 7E and 7F), which was consistent with increased levels of BCL2 protein in HOXA9-expressing subcutaneous tumor (Figure 4F). In order to confirm the link between HOXA9-mediated BCL2 overexpression and resistance to temozolomide, and to explore novel combinatorial treatments to GBM, we evaluated how ABT-737, a potent small molecular inhibitor of BCL2 family proteins, affects the response of U87MG cells to temozolomide. While no significant cytotoxic effects were observed for two different doses of ABT-737 alone (0.25 and 5 μM), the combination of ABT-737 with temozolomide significantly increased cytotoxicity of U87MG cells, an effect that was significantly more pronounced in *HOXA9*-positive cells (Figure 7G). This finding supports the evidence that BCL2 could be associated with temozolomide resistance in U87MG *HOXA9*-positive cells. Importantly, the use of this BCL2 inhibitor together with temozolomide did not affect cytotoxicity of cells lacking BCL2 expression (Supplementary Figure 14A and 14B), which supports the specificity of the observed effect. Importantly, in the TCGA dataset, 87% of the *HOXA9*-high GBM patients also display high levels of *BCL2*, emphasizing that the link between HOXA9 and BCL2 is not only present in *in vitro* and *in vivo* models of GBM, but is also relevant in the clinical setting. These data supports the future exploitation of BCL2 inhibitors combined with temozolomide for the treatment of GBM patients.

## DISCUSSION

Previous studies have shown that *HOX* genes are part of large gene expression signatures that associate with glioblastoma self-renewal and therapy response [12–14]. However, the specific contribution of individual *HOX* genes in glioblastoma behavior has been largely unknown. In particular, *HOXA10* expression was recently shown to be regulated by the Trithorax protein mixed lineage leukemia (MLL), resulting in the activation of HOXA10-target genes that contribute to the tumorigenic potential of glioblastoma stem cells [14]. Another study suggested *HOXA10* may contribute to temozolomide resistance *in vitro* [15]. While HOXA9 was also previously shown to display pro-viability and anti-apoptotic functions in glioblastoma *in vitro* [16], the downstream functional consequences of *HOXA9* activation have remained unknown. Here, we specifically investigated for the first time the genome-wide transcriptional targets of HOXA9 in four independent GBM cell models, and evaluated how HOXA9 affects critical cellular and molecular processes of the malignant phenotype of gliomas, using a combination of *in vitro* and *in vivo* models.

Interestingly, our expression array data showed an HOXA9-mediated enrichment of genes involved in several important hallmarks of cancer (Figure 2 and Supplementary Figure 3). The fact that HOXA9 transcriptomes were so distinct within GBM cells (U87MG, U251 and GBML18) and in immortalized astrocytes (hTERT/E6/E7) cells suggests that HOXA9 targets different genes in a cell-type dependent manner, which is in accordance with previous reports in leukemia models [19]. Despite these specificities, GSEA analysis revealed that *HOXA9* transcriptomes are enriched for genes involved in stem cell signatures, which fits well with the previously reported *HOX*-dominated gene expression signatures found in glioblastoma stem cells [12–14, 19], and suggests HOXA9 as a critical component of that signature. Concordantly, the single overexpression of HOXA9 increased the capacity of these cells to grow as neurospheres and upregulated the expression of Nestin (Figure 3), both of which have been related to the stemness potential of GBM cells [25]. Our data in GBM also identified novel HOXA9 target genes involved in cell proliferation and cytoskeletal organization pathways, which have previously been identified as HOXA9 downstream effectors in leukemia [19]. Genes involved in other critical pathways, including DNA repair, cell invasion, cell mobility and immune response, were also found up-regulated due to HOXA9 (Figure 2, Supplementary Figure 3 and Supplementary Figure 10). Together, our findings suggest that several molecular pathways act downstream of HOXA9 to maintain a malignant phenotype in GBM. Critically, these HOXA9-driven signatures may be pharmacological reverted by PI3K inhibitors and chromatin-remodeling drugs, as suggested previously [13, 16, 33] and now by our connectivity map analyses (Supplementary Figure 3H).

The importance of the cancer stem cell population in tumor initiation, progression, and response to therapy has been well documented [34]. Consistent with the increased stem cell features displayed by *HOXA9*-positive cells, our subcutaneous xenograft models using U87MG cells showed that *HOXA9* accelerates tumor growth and tumor-related angiogenesis, with a concomitant increase in the expression of proteins involved in these cellular processes (Cyclin D1, Ki-67, BCL2, and PECAM1; Figure 4). Strikingly, we also showed that hTERT/E6/E7 immortalized astrocytes that overexpress *HOXA9* are able to originate orthotopic gliomas *in vivo*, in contrast to *HOXA9*-negative hTERT/E6/E7 cells (Figure 5), which may partly be explained by the notion that tumor-initiating cells have high stem cell potential. While HOXA9 is an established oncogene in the context of leukemia [35, 36], our study is the first to clearly demonstrate the oncogenic potential of HOXA9 in gliomas.

Previous studies have provided circumstantial evidence suggesting *HOX* genes may be important in the aggressiveness of GBM [11–13, 16]. However, the functional mechanisms by which particular *HOX* genes may contribute to this malignant phenotype have not been elucidated. In the present study, we employed both overexpression and shRNA-mediated silencing approaches to investigate the functional roles of HOXA9 in four independent GBM models. Globally, our data show that HOXA9 increases cell viability, invasion, and resistance to cell death in GBM (Figure 6 and Supplementary Figure 11), establishing a good parallel with HOXA9 functions observed in leukemia models [37, 38]. In addition, HOXA9 significantly increased the resistance of all tested GBM cell lines to temozolomide treatment (Figure 6 and Supplementary Figure 11), establishing HOXA9 as a new biomarker of temozolomide response in GBM. Of note, this HOXA9-driven resistance to temozolomide is likely to be independent of p53 function, as the cell lines tested here have different p53 status ( p53 wild-type in U87MG; p53 mutant in U251; p53 inactivated in hTERT/E6/E7). Critically, the increased resistance to temozolomide mediated by HOXA9 was validated *in vivo*, as U87MG-HOXA9-xenografted animals presented a significantly shorter survival than those xenografted with U87MG-MSCV cells (Figure 7). In order to provide further insights into the mechanisms of HOXA9-mediated resistance to chemotherapy, which are critical to help designing novel therapeutic strategies to treat GBM patients, we investigated canonical markers of temozolomide response in GBM. Interestingly, HOXA9 did not affect the protein levels of MGMT, or the DNA methylation status of the *MGMT* promoter, which is a major regulator of temozolomide response. In contrast, HOXA9 affected the expression of other key proteins involved in temozolomide response, including down-regulation of MMR family proteins PMS2 and MSH6 (Figure 7). MMR deficiency has been implicated in preclinical studies as an additional mechanism of resistance to alkylating agents, including temozolomide [39]. Specifically, reduction of PMS2 protein levels was found to be associated with temozolomide resistance, and inactivating mutations of *MSH6* gene and loss of MSH6 function have been associated with emergent temozolomide resistance in GBM patients [40, 41]. Our data suggest a novel HOXA9-driven mechanism of MMR deficiency in GBM that may decrease the therapeutic value of temozolomide. Since reestablishing MMR protein levels/function is not clinically feasible, we explored additional mechanisms by which HOXA9 may drive temozolomide resistance in GBM. Interestingly, we found that HOXA9 increases the expression of the anti-apoptotic protein BCL2 *in vitro* and *in vivo* (microarray, qPCR, immunohistochemistry and Western blot data). Importantly, the majority of GBM patients with *HOXA9*-high tumors also display high levels of *BCL2* (TCGA data), indicating that this molecular link is clinically relevant and not only exclusive of GBM models. Indeed, BCL2 was recently shown to be a target of HOXA9 in acute myeloid leukemia [42]. BCL2 has been considered an attractive target for anticancer therapies as it can be pharmacologically inhibited and, thus, potentiate apoptosis in cancer cells [30–32]. Indeed, the blockade of BCL2 activity has shown promising results in several cancers, such as pediatric lymphoblastic leukemia [43], neuroblastoma [44], melanoma [45], genitourinary neoplasm [30], and glioblastoma [46]. A recent study showed that the down-regulation of BCL2 restored temozolomide sensitization in a GBM cell line [47]. Another study showed a positive effect of ABT-737, a BCL2 inhibitor, in restoring chemosensitivity to vincristine and etoposide [46]. These data prompted us to evaluate how inhibition of BCL2 function could sensitize *HOXA9*-positive GBM cells to temozolomide. Interestingly, a combination of temozolomide and ABT-737 had a significant increased cytotoxic effect as compared to temozolomide alone, exclusively in U87MG-HOXA9 cells (Figure 7). Of note, a similar combination of temozolomide and ABT-737 did not affect the viability of BCL2-negative cells, providing a proof-of-concept that the HOXA9-mediated resistance to temozolomide can be partly reverted by specifically inhibiting BCL2 function. Of note, ABT-737 may be too large to cross the blood-brain barrier, which could limit its clinical application. Nevertheless, malignant gliomas frequently present a focal breach of the BBB caused by a loss of tight junctions [48], allowing blood-borne molecules to enter the brain parenchyma. On other hand, the intracranial delivery of ABT-737 might be possible in the future through the use of nanoparticle-based delivery systems, through intraventricular administration, or by using implantable wafers. Future studies should further investigate how these combinatory treatments can be more effective to treat the subset of GBM patients with high levels of HOXA9/BCL2, helping to improve the outcome of these highly therapy resistant tumors.

In conclusion, our study establishes *HOXA9* as a critical oncogene in the initiation and progression of glioma, and provides evidences into the mechanisms by which patients with HOXA9-positive GBMs respond poorly to temozolomide and have worse clinical outcomes. These findings may have clinical impact by allowing the timely identification of patients that may not benefit from temozolomide-based treatment, who could be redirected to alternative therapeutic regimens, namely based on BCL2 inhibition.

## MATERIALS AND METHODS

Detailed methodologies are described in Supplementary Data.

### TCGA and REMBRANDT meta-analysis in glioma patients

Gene expression, copy number, DNA methylation, *MGMT* promoter methylation, GBM subtype classification, and clinical data from GBM, lower-grade glioma (LGG, WHO grades II and III), and normal samples were collected from TCGA (http://tcga-data.nci.nih.gov/). *HOXA9* expression and prognostic value was also assessed in patients from REMBRANDT (https://caintegrator.nci.nih.gov/rembrandt/).

### Cell lines

Four different GBM cell line models were used: two established human GBM cell lines (U87MG and U251), immortalized human astrocytes (hTERT/E6/E7), and a primary GBM culture (GBML18) established in our lab as previously described [49]. All cell lines were maintained in Dulbecco's Modified Eagle Medium (DMEM) supplemented with 10% FBS and 1% penicillin-streptomycin and incubated at 37°C in 5% CO_2_. The commercial cell lines U87MG and U251 were authenticated by short tandem repeat profiling.

### HOXA9-overexpression and silencing in GBM cells

U87MG and hTERT/E6/E7 were previously retrovirally infected with MSCVneo vectors containing *HOXA9* cDNA [16]. For *HOXA9* silencing**,** GBML18 and U251 cells were transfected with pGFP-V-RS plasmid containing HOXA9-specific shRNAs or non-effective shRNAs sequences (Origene).

### Gene expression microarray analyses

Genome-wide transcriptome profiles of U87MG and hTERT/E6/E7 cells with MSCV (control) or HOXA9 vectors, and U251 and GBML18 with pGFP-V-RS plasmid containing HOXA9-specific shRNAs or non-effective shRNAs sequences were performed by expression microarrays (Whole Human Genome 44k Oligo Microarray Kit, Agilent) and validated by RT-PCR or quantitative real-time PCR. Microarray data and full details on the experimental procedures are published online at Gene Expression Omnibus (http://www.ncbi.nlm.nih.gov/geo/query/acc.cgi?token=ebmfagkwxrkndqx&acc; GEO accession number GSE56517).

Differentially-expressed genes were used for Database for Annotation, Visualization and Integrated Discovery (DAVID) analyses and displayed in KEGG, GO, or Reactome pathways. For gene set enrichment analysis (GSEA; http://www.broad.mit.edu/gsea/), databases from MSigDB C2 collection version three were used, and results with false discovery rate (FDR) < 0.25 were considered significant [50]. Connectivity Map was used to verify the similarity between HOXA9 transcriptome in GBM models with drug treatments-induced gene expression signatures in cancer models [51].

### Functional assays

Neurospheres formation assay was performed in hTERT/E6/E7 and U87MG cell lines. For all cell lines the half maximal inhibitory concentration (IC_50_) of temozolomide (TMZ) was determined by MTS (Promega), following the manufacturer´s instructions. Time-course cell viability assays were determined by Trypan blue exclusion assays (Gibco^®^) and MTS tests. Cell death was evaluated by annexin V staining (BD Biosciences), followed by flow cytometry analyses. Invasion was measured using the BD BioCoat™ Tumor Invasion System (BD Biosciences) as indicated by the manufacturer's instructions.

### Immunohistochemistry, immunofluorescence and western blot

Immunohistochemistry or immunofluorescence staining was conducted using antibodies against Cyclin D1, Ki-67, BCL2, Nestin and PECAM1. Tumor vascular area was calculated as the percentage of PECAM1 positive cells in at least 17 consecutive areas (100x: ~0.63 mm^2^) evaluated per tumor. Western blot was performed using antibodies against MGMT, BCL2, MSH3, MSH2, PMS2, MSH6, MLH3, MLH1, PCNA, PARP1, APEX1, XRCC1. All antibodies are listed in Supplementary Table 2.

### RT-PCR and quantitative real-time PCR

RNA extraction was performed by TRIzol (Invitrogen) and cDNA synthetized using RT-Phusion Kit, Thermo Scientific. Gene-specific mRNA levels were assessed by standard RT-PCR or quantitative PCR (qPCR), using the ΔΔCt method as described previously [52]. All primers sequences are listed in Supplementary Table 3.

### DNA isolation, bisulfite treatment and *MGMT* methylation-specific PCR

DNA was isolated from cell lines using TRIzol (Invitrogen) and treated with sodium bisulfite using the EZ DNA Methylation-Gold^TM^ Kit (Zymo Research), according to the manufacturer's protocol. Methylation-specific PCR for *MGMT* promoter was performed as previously described [53].

### *In vivo* GBM xenografts

All experiments with mice were approved by institutional and national ethical committees (Direção Geral de Alimentação e Veterinária, Portugal) and in accordance with European Union Directive 2010/63/EU. Subcutaneous and orthotopic xenograft models were performed by injecting U87MG and hTERT/E6/E7 cells with or without HOXA9.

### Statistical analyses

SPSS 19.0 software (SPSS, Inc.), GraphPad Prism software (version 5.0) and the Bioconductor platform were used. Statistical significance was considered when *p* < 0.05.

## SUPPLEMENTARY MATERIALS AND METHODS


