# Supplementary material for: A transcriptomic signature mediated by HOXA9 promotes human glioblastoma initiation, aggressiveness and resistance to temozolomide
Source: Oncotarget. 2015 Feb 20;6(10):7657–74. doi: 10.18632/oncotarget.3150 (PMC4480707; doi:10.18632/oncotarget.3150)
Supplement: Supplementary file 1 [file oncotarget-06-7657-s001.pdf]

SUPPLEMENTARY MATERIALS AND METHODS

TCGA data meta-analysis in glioma patients

The Cancer Genome Atlas (TCGA) provides comprehensive and systematic genomic characterization and sequence analysis of gliomas, including information about gene expression, copy number alterations, and DNA methylation status, as well as clinical information from most patients [1]. This dataset included 572 glioblastomas (GBM), 27 lower-grade gliomas (LGG, WHO grades II-III) and 10 unmatched normal samples.

Gene expression data was hybridized using Agilent G4502A 244K by the University of North Carolina, Lineberger Comprehensive Cancer Center. For gene expression analyses, probes values for *HOXA9* (A_23_P500998), *MGMT* (A_23_P104323) and *BCL2* (A_23_P352266 and A_24_P512775) were pre-processed and normalized according to "Level 3" specifications of TCGA (see http://cancergenome.nih.gov/dataportal/ for details). *HOXA9* expression values were categorized as high expression for TCGA "Level 3" values ≥ 3. *MGMT* and *BCL2* expression values were categorized as high expression for TCGA "Level 3" values > 0. Clinical data of each patient included in TCGA was provided by the Biospecimen Core Resources (BCRs), and includes information about the patient age at diagnosis, gender, Karnofsky performance status (KPS), and overall survival (days to death or to last follow-up). These data are available for download through TCGA data matrix [1] (http://tcga-data.nci.nih.gov/tcga/dataAccessMatrix.htm).

Gene copy number data was assessed in 372 GBM samples by the Eli and Edythe L. Broad Institute of the Massachusetts Institute of Technology (MIT) and Harvard University. The Affymetrix Genome-Wide Human SNP Array 6.0 was used. Log_2 copy number tumor/normal ≥ 0.5 (gene copy number ≥ 3) was considered for gene amplification and ≤ -0.5 (gene copy number ≤ 1) for gene deletion.

Illumina Infinium Human DNA Methylation 450 array was used by the Johns Hopkins University and the University of Southern California joint group to evaluate tumor DNA methylation of 74 GBM samples. In our study, the methylation of all 25 probes that hit *HOXA9* (cg01891966 to cg02643054) were evaluated. *MGMT* promoter methylation status and subtype classification of TCGA GBM samples were obtained at https://tcga-data.nci.nih.gov/docs/publications/gbm_exp/.

REpository for Molecular BRAin Neoplasia DaTa (REMBRANDT) analyses

The REMBRANDT platform (https://caintegrator.nci.nih.gov/rembrandt/) [2] was used to evaluate *HOXA9* expression and its prognostic value in an independent set of 181 GBM patients. *HOXA9* expression was categorized as high for fold-change values > 4 compared to normal samples (corresponding to 95 of 181 patients).

Cell culture

All cell lines were cultured in Dulbecco's Modified Eagle Medium (DMEM, Gibco, Invitrogen), supplemented with 10% fetal bovine serum (FBS) and 1% penicillin and streptomycin. Incubations were performed at 37ºC in a humidified atmosphere containing 5% CO_2. To select the retrovirally infected U87MG and hTERT/E6/E7 cells (previously established by [3]), G418 (500 µg/mL) was used since these constructs contain the neomycin resistance gene.

HOXA9-silencing by shRNA in GBM cells

GBML18 and U251 cells were plated at 1×10^5 cells/well in 24-well plates. Cells were transfected with *HOXA9* gene-specific shRNA sequences in pGFP-V-RS plasmid (TG307647, clones GI330583 and GI330584, Origene) or a non-efective shRNA cassette in pGFP-V-RS plasmid (scrambled negative control; TR30013, Origene). For transfection, Polyjet reagent (SignaGen Laboratories) was used according to the manufacturer's protocol (ratio plasmid/polyjet, 1:3). Selection of transfected cells was performed with puromycin (0.5 µg/mL), since these constructs present the puromycin resistance gene.

Gene expression microarray experiments

Total RNA was purified with Trizol (Invitrogen) from respectively four or three independent experimental replicates of *HOXA9*-overexpressing and –silenced cell lines (hTERT/E6/E7-MSCV, hTERT/E6/E7-HOXA9, U87MG-MSCV, U87MG-HOXA9, U251-shControl, U251-shHOXA9, GBML18-shControl, GBML18-shHOXA9), totalizing 28 microarray analyses. After RNA quality control by RNA Bioanalyzer (Agilent) analyses, the replicates of each cell line were used for cDNA synthesis and labeling, cRNA synthesis and amplification, and hybridization to Human Whole Genome 44K Kit (G2534A, Agilent) microarrays, followed by slide scanning and feature extractions, according to the manufacturer's instructions (One-Color Microarray-Based Gene Expression Analysis, Agilent Technologies).

Microarray data validation by RT-PCR

Upon identification of HOXA9-mediated differentially expressed genes in each cell line by microarray data analysis (see statistical methods

for details), confirmatory reverse-transcriptase PCR (RT-PCR or qPCR) analyses were performed in a subset of target genes to validate the microarray data. Specifically, genes among the most differentially-expressed were selected and tested by RT-PCR or qPCR in each cell line (*RAC2*, *CXCL1*, *NDRG1*, and *TOX2* for hTERT/E6/E7 cells; *ICAM2*, *BAMBI*, *ANGPT2*, and *PDGFRB* for U87MG cells; *TOX2, NDRG1, RAC2* and *NPR3* for GBML18 cells; *C10orf10, PDGFRβ, DKK1* and *SOX2* for U251 cells). See Supplementary Table 3 for information about PCR parameters and primer sequences.

Connectivity map analyses

The Connectivity Map tool [4] was used to search for drug treatments-induced gene expression signatures that may be similar to the *HOXA9* transcriptomic signatures obtained in the microarray data derived from hTERT/E6/E7, U87MG, U251 and GBML18 cells. The 100 genes with lowest adjusted *p*-value (50 up-regulated and 50 down-regulated) in HOXA9-overexpressing (U87MG-HOXA9 and hTERT/E6/E7-HOXA9) or HOXA9-silenced (U251-shHOXA9 and GBML18-shHOXA9) cells, as compared to their respective control counterparts (U87MG-MSCV, hTERT/E6/E7-MSCV, U251-shControl and GBML18-shControl), were used as a signature to query the Connectivity Map.

Neurosphere formation assay

Cell lines were plated at low density (500 cells/mL) in NeuroCult NS-A Proliferation Kit (Human) supplemented with 20 ng/mL EGF, 20 ng/mL b-FGF, 20 ng/mL heparin, 2 mM glutamine, and 1% B27 (all from Life Technologies). Cells were incubated for up to 8–15 days. Pictures were taken and quantification was performed by ImageJ software (version 1.44).

Immunohistochemistry and immunofluorescence

Tissues sections were deparaffinized and rehydrated by xylene and ethanol series. Immunohistochemical staining was performed using the LabVision kit (UltraVision Large Volume Detection System Anti-polyvalent, HRP) according to the manufacturer's instructions. For PECAM1 staining, the immunohistochemical protocol was performed using a biotinylated horse anti-goat antibody. For all stainings DAB substrate (DAKO) was used as chromogen, followed by counterstaining with hematoxylin. For immunofluorescence, cells were fixed with Paraformaldehyde (PFA) 4% for 15 minutes and incubated with the primary antibody against Nestin. Nuclei were stained with DAPI (VECTASHIELD® Mounting Medium with DAPI, Vector Laboratories). All antibodies used and their respective concentrations are listed in Supplementary Table 2.

Cell viability assays

For half-maximal inhibitory concentration (IC_{50}) of TMZ, 4×10^3 cells were plated in 24-well plates, and exposed to different doses of TMZ (10, 25, 50, 100, 200, 500, 1000 µM) for 5 days. Cell viability was determined by MTS, and IC_{50} values were calculated by a nonlinear regression (curve Fit) based on sigmoidal dose-response (variable slope) using GraphPad Prism 5.0 (GraphPad software, Inc.).

Cell viability under basal conditions and after exposure to temozolomide (TMZ, Sigma-Aldrich, dissolved in DMSO) was determined by Trypan blue (Gibco®) exclusion assay and MTS (Promega) tests. For the Trypan blue assay, cells were plated at an initial density of 1.5×10^4 cells/well in 6-well plates, in triplicates, and incubated at 37ºC (5% CO_2). After 24 hours, hTERT/E6/E7, U87MG, U251 and GBML18 with differential levels of *HOXA9*, were treated with TMZ (600 µM, 600 µM, 10 µM and 50 µM, respectively) or vehicle (1% DMSO) in DMEM medium supplemented with 10% FBS for 9 days. Culture medium containing TMZ or vehicle was renewed every 3 days. At each timepoint (1–9 days), total cells were trypsinized and the suspension mixed with trypan blue (1:1 ratio). Viable cells were counted in triplicates under the microscope using hemocytometers. For MTS assays, cells were plated at an initial density of $4\text{x}10^3$ cells/well in 24-well plates, in triplicates. After 24 hours, cells were treated with DMEM supplemented with 10% FBS and drugs (TMZ and/or ABT-737, Selleck Chemicals) or vehicle (1% DMSO), and incubated for 2, 4, and 6 days (medium with drugs or vehicle was renewed every 3 days). At each time point, cells were exposed to medium containing MTS in a 5:1 ratio for 2 h in a humidified atmosphere at 37°C and 5% CO_2, and the optical density was determined at 490 nm.

Cell death

Cell lines hTERT/E6/E7, U87MG, U251 and GBML18, with differential levels of *HOXA9*, were plated at an initial density of 7.5×10^4 cells for hTERT/E6/E7, and for the remaining cell lines 1.0×10^5 cells, per T25 flask in 3 mL of DMEM supplemented with 10% FBS. After 24 hours, cells were treated with TMZ (600 µM, 600 µM, 10 µM and 50 µM, respectively) or vehicle (1% DMSO). Cell death was evaluated after 5 days of treatment by annexin V-fitc or annexin V-alexa fluor 647, according to the manufacturer's instructions (BD Biosciences), followed by flow cytometry analyses. A total of at least 10,000 events were acquired by flow cytometer. The results were analyzed by FlowJo software (version 7.6).

Cell invasion

Invasion was measured using the BD BioCoat™ Tumor Invasion System (BD Biosciences) as indicated by the manufacturer. Briefly, cell lines were seeded in triplicate into the apical side of Matrigel coated chambers at an initial density of 5×10^4 cells/mL in 500 µL DMEM with 5% FBS, with TMZ or vehicle (1% DMSO). The lower chambers were filled with 500 µL of DMEM containing 10% FBS and 20 ng/mL EGF as a chemoattractant. After 48 h of incubation, cells that invaded through Matrigel to the membrane were stained with DAPI (1:1000, Vector Laboratories) and scanned using an inverted fluorescent microscope. To quantify the number of invading cells, 10 pictures at 20x-magnification were acquired per well, and the total cell numbers were obtained using ImageJ software (version 1.44).

Western blot assays

Cells were trypsinized, washed with PBS and lysed for 1 hour at 4ºC using a lysis buffer containing 50 mM Tris-HCL, 5 mM EDTA, 150 mM NaCl, 1% NP-40, 1 mM PMSF and inhibitors of proteases 1x (Roche Diagnostics). Protein concentration was determined by the Bradford method. Using a 10% SDS-polyacrylamide gel, the total protein extracts (50 µg/lane) were separated by electrophoresis and transferred to nitrocellulose membranes (GE Healthcare, Life Sciences). The immunodetection was achieved using antibodies for human MGMT, MLH1, MLH3, PCNA, α-tubulin, MSH2, MSH3, MSH6, PMS2, PARP1/2, XRCC1, BCL2, and APE1 (Supplementary Table 2). Blots were revealed with peroxidase-conjugated secondary anti-rabbit or anti-mouse antibodies (Supplementary Table 2) followed by ECL chemiluminescence solution (SuperSignal West Femto Chemiluminescent Substrate; Thermo Scientific). Protein expression quantification was performed by ImageJ software (version 1.44).

In vivo GBM xenografts

For subcutaneous models, a total of $2\text{x}10^6$ cells (hTERT/E6/E7-MSCV, hTERT/E6/E7-HOXA9, U87MG-MSCV and U87MG-HOXA9) were injected into the right flank of nude mice (8-weeks old athymic nude Foxn1^{nu} male mice, from Harlan Laboratories). Tumor size and body weight were measured every 3 days. Tumor volume was calculated by assessing the two largest sides (v = (3.14xL1xL1xL2)/6). After euthanasia, tumors were divided for molecular analyses (frozen samples) or for immunohistochemical analyses, for which samples were fixed by immersion in formalin and subsequently embedded in paraffin.

For intracranial orthotopic models, a total of 2×10^5 cells (hTERT/E6/E7-MSCV, hTERT/E6/E7-HOXA9, U87MG-MSCV and U87MG-HOXA9) were stereotactically injected into the brain striatum (1.8 mm right, 0.4 mm back, and 2.5 mm deep from the bregma) of 8-weeks old athymic nude Foxn1^{nu} male mice. Mice injected with U87MG cells were randomly assigned to control or treatment groups (8 mice/group) 2 weeks after injection. TMZ treatment was administered by oral gavage with a daily dosage of 50 mg/kg diluted in saline solution, over 2 cycles of 21 days (5 days on and 2 days off). Animals' body weight was evaluated 3 times per week, and general behavior and symptomatology daily. Humane endpoints for sacrifice were established as severe weight loss (>20%), neurological dysfunction, seizures, or moribund condition. All brains were collected for histological and molecular analyses.

Statistical analyses

The effect of *HOXA9* in the overall survival of GBM patients from TCGA was represented by Kaplan-Meier survival curves, and the differences evaluated by univariate (Log-rank test) and multivariate survival analysis (Cox proportional hazard model, adjusted for the potential confounding effect of other putative prognostic factors, including patient age, gender, KPS, *MGMT* methylation status and treatment with chemotherapy). These analyses were made with SPSS 19.0 software (SPSS, Inc.).

For gene expression microarray data processing and analysis, the *limma* package [5] of the Bioconductor software platform (http://www.bioconductor.org) was used. Data was first pre-processed performing background correction (using the *normexp* method), normalized between arrays using quantile normalization and $\log_2$-transformed. Probes representing control spots were filtered, keeping only the ones with status equal to "Gene". Replicated probes (with the same *ProbeName*) were averaged. Genes consistently differentially expressed across quadriplicates for each paired cell line (hTERT/E6/E7-MSCV *vs.* hTERT/E6/E7-HOXA9; U87MG-MSCV *vs.* U87MG-HOXA9, U251-shControl *vs.* U251-shHOXA9, and GBML18-shControl *vs.* GBML18-shHOXA9) were identified by fitting linear models for each gene, followed by the calculation of the relevant statistics using the Empirical Bayes method as implemented by the *limma* Bioconductor package [6]. Genes were ranked according to their adjusted *p*-values, where false positive rates were assessed to address the issues related to multiple testing.

For *in vitro* tests, a repeated measures ANOVA was used to assess differences in Trypan blue viability curves. For MTS viability assay, cell death, cell invasion, cell migration, neurospheres formation, and levels of protein

and gene expression, *t*-tests were used to assess statistical differences.

Overall survival of orthotopic GBM xenografted mice was compared between groups (*HOXA9*-positive versus *HOXA9*-negative, with or without TMZ treatment) by the log-rank test, and plotted as Kaplan-Meier curves.

Spearman correlation was used to evaluate the association between the expression levels of *HOXA9* in the subcutaneous tumor models and the final tumor volume.

All statistical tests were two-sided, and significance was considered when $p < 0.05$.

REFERENCES

1. TCGA: Comprehensive genomic characterization defines human glioblastoma genes and core pathway. Nature. 2008; 455:1061–1068.
2. National Cancer Institute REMBRANDT. home page: http://rembrandt.nci.nih.gov. 2005.
3. Costa BM, Smith JS, Chen Y, Chen J, Phillips HS, Aldape KD, Zardo G, Nigro J, James CD, Fridlyand J, Reis RM, Costello JF. Reversing HOXA9 oncogene activation by PI3K inhibition: epigenetic mechanism and prognostic significance in human glioblastoma. Cancer Res. 2010; 70:453–462.
4. Lamb J, Crawford ED, Peck D, Modell JW, Blat IC, Wrobel MJ, Lerner J, Brunet JP, Subramanian A, Ross KN, Reich M, Hieronymus H, Wei G, Armstrong SA, Haggarty SJ, Clemons PA, et al. The Connectivity Map: using gene-expression signatures to connect small molecules, genes, and disease. Science. 2006; 313:1929–1935.
5. Smyth GK. Limma: linear models for microarray data. Bioinformatics and computational biology solutions using R and Bioconductor. Springer 2005: 397–420.
6. Smyth GK. Linear models and empirical bayes methods for assessing differential expression in microarray experiments. Statistical applications in genetics and molecular biology. 2004; 3:Article3.

SUPPLEMENTARY FIGURES AND TABLES

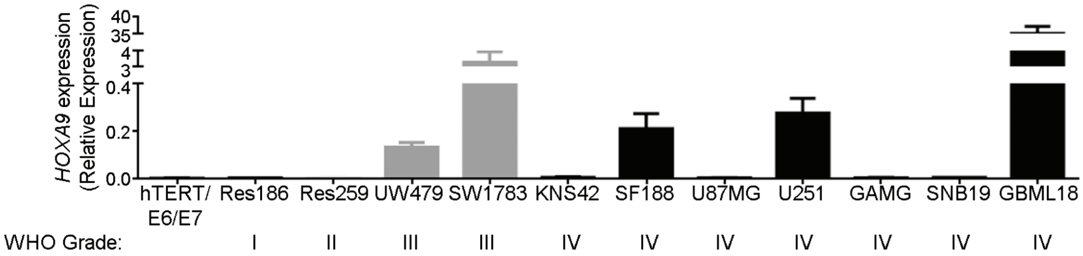

Supplementary Figure 1: Quantification of endogenous *HOXA9* mRNA levels in hTERT/E6/E7 immortalized astrocytes and in a panel of glioma cell lines (WHO grades I–IV). hTERT/E6/E7 and U87MG cells did not present detectable levels of HOXA9 and were used for overexpression approaches. U251 and GBML18 cell lines are the adult GBM cell lines with higher expression of *HOXA9*, which were later used for shRNA-mediated silencing of HOXA9.

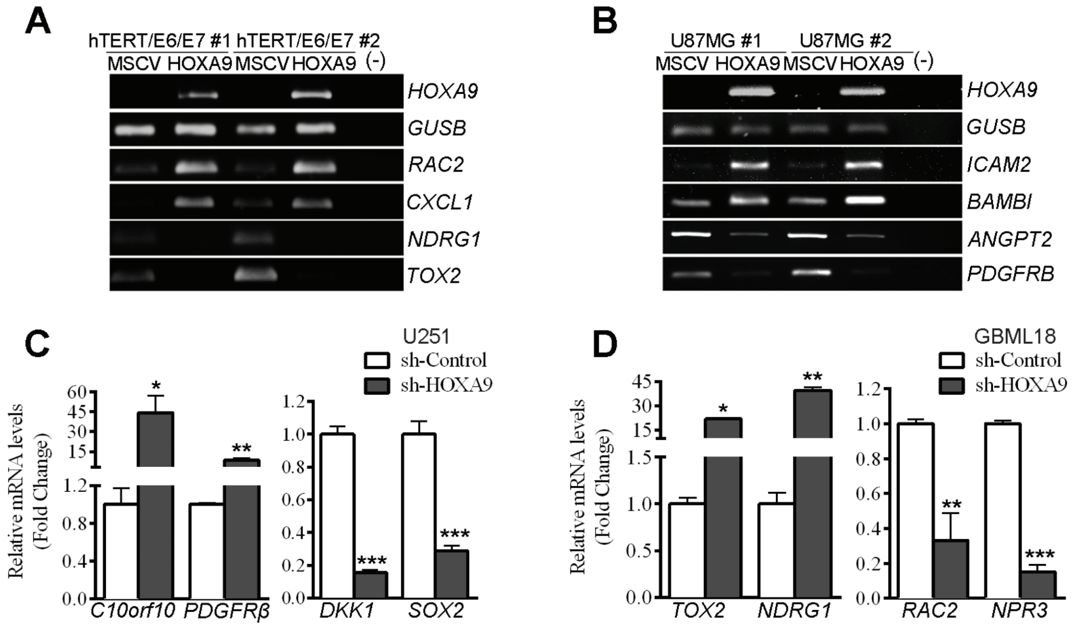

Supplementary Figure 2: Validation of the HOXA9 transcriptome in hTERT/E6/E7 human immortalized astrocytes, and U87MG, U251, and GBML18 glioblastoma cells. Semi-quantitative RT-PCR **(A)** and **(B)** and q-PCR **(C)** and **(D)** were performed for several genes identified as differentially expressed in the microarray data; representative results are shown for two genes up- and two genes down-regulated due to *HOXA9*-levels manipulation in each cell line.

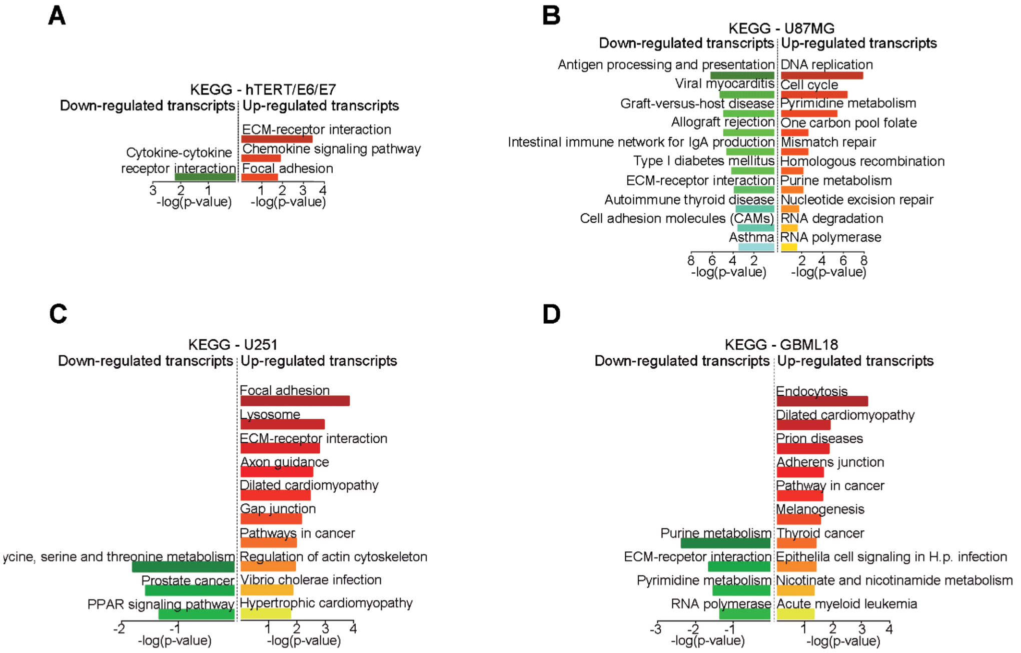

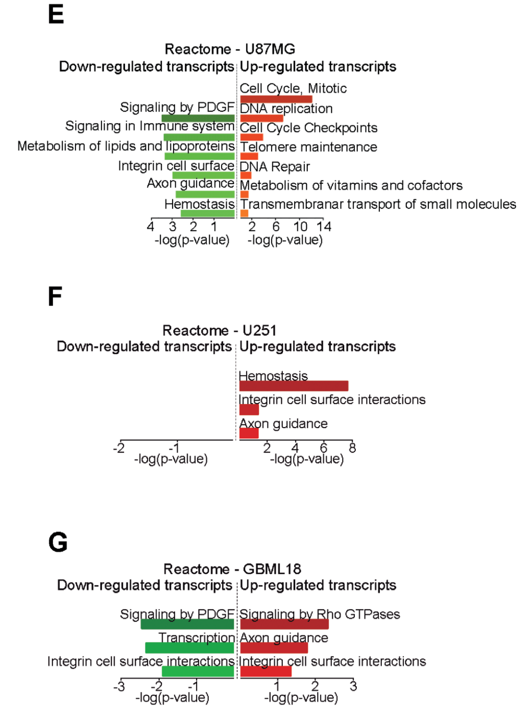

H

	Rank in output	Cmap name	Cell line	n	Enrichment	*p* -value	Specificity
hTERT/E6/E7	1	Tanespimycin	MCF7	36	0.372	<0.0001	0.3842
	15	Geldanamycin	MCF7	10	0.514	0.006	0.3129
	23	Monorden	MCF7	12	0.457	0.008	0.2216
U87-MG	2	LY-294002	PC3	12	-0.633	<0.0001	0.0769
	4	Tanespimycin	MCF7	36	-0.529	<0.0001	0.123
	5	Trichostatin A	MCF7	92	-0.524	<0.0001	0.2871
	6	LY-294002	MCF7	34	-0.427	<0.0001	0.1875
	8	Vorinostat	MCF7	7	-0.721	<0.001	0.233
	9	Tanespimycin	HL60	12	-0.579	<0.001	0.1786
	12	Sirolimus	MCF7	25	-0.402	<0.001	0.3425
	13	Alvespimycin	MCF7	7	-0.702	<0.001	0.0234
	14	Monorden	MCF7	12	-0.536	0.001	0.0368
	23	Ellipticine	MCF7	2	-0.951	0.005	0.097
	24	Sirolimus	PC3	8	-0.569	0.006	0.2676
	27	Wortmannin	MCF7	10	-0.501	0.007	0.277
	29	MS-275	PC3	2	-0.94	0.008	0.1482
U251	17	Sirolimus	PC3	8	0.567	0.006	0.2177
	19	Paclitaxel	PC3	2	-0.948	0.006	0.016
GBML18	1	Trichostatin A	PC3	55	0.372	<0.0001	0.7511
	8	Etoposide	MCF7	2	-0.952	0.005	0.025
	11	Semustine	PC3	2	-0.946	0.006	0.0211
	17	Finasteride	PC3	2	-0.936	0.009	0.0182

Cmap: Connectivity Map; n: Number of treatments and vehicle pair

Supplementary Figure 3: Genome-wide characterization of HOXA9 transcriptomes in hTERT/E6/E7 human immortalized astrocytes, and U87MG, U251 and GBML18 glioblastoma cells. (A–G) DAVID was used to query the HOXA9-transcriptome from each cell line (A, hTERT/E6/E7; B and E, U87MG; C and F, U251; and D and G, GBML18), in order to identify enriched biological terms on the differentially expressed genes extracted from the microarray data. Statistically significant enriched KEGG pathways **(A–D)**, and Reactome pathways **(E–G)** are shown. **(H)** Connectivity Map analysis for drug treatments in cell lines that induce gene expression signatures similar to the ones obtained in the HOXA9 microarray data from hTERT/E6/E7, U87MG, U251 and GBML18 cells. The top 100 of the genes with lowest adjusted *p*-value (50 most up-regulated and 50 most down-regulated) in HOXA9-overexpressing (U87MG-HOXA9 and hTERT/E6/E7-HOXA9) or HOXA9-silenced (U251-shHOXA9 and GBML18-shHOXA9) cells, as compared to their respective control cells (U87MG-MSCV, hTERT/E6/E7-MSCV, U251-shControl and GBML18-shControl) were used. Examples of drugs that have been tested for cancer therapy and evaluated in cancer cell lines are presented, including PI3K inhibitors (LY-294002, Sirolimus, Wortmannin), histone deacetylase inhibitors (Trichostatin A, Vorinostat, MS-275), and HSP90 inhibitors (Tanespimycin, Geldanamycin, Monorden, Alvespimycin).

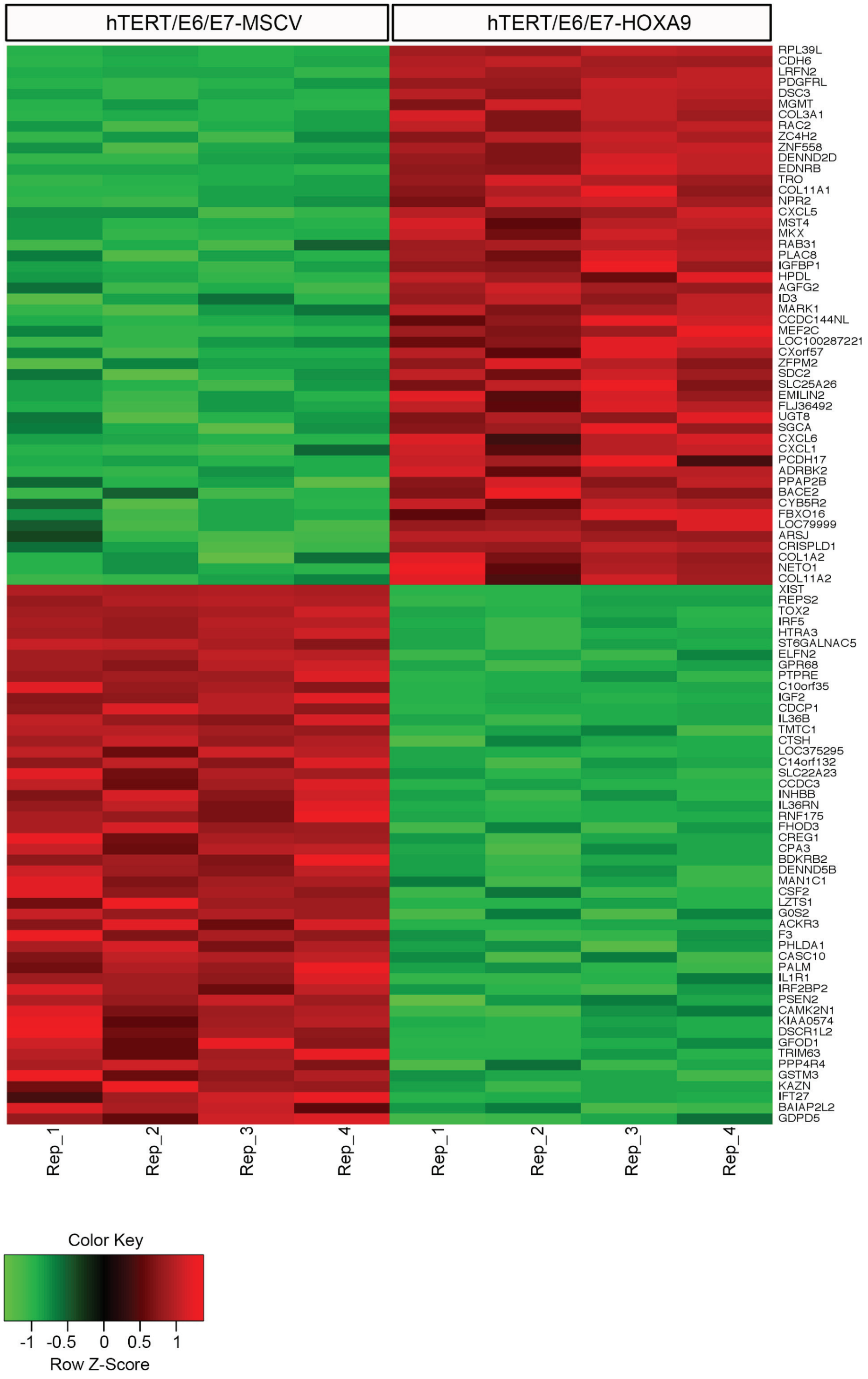

Supplementary Figure 4: Heatmap representation of the top 50 most up-regulated and top 50 most down-regulated genes in hTERT/E6/E7-HOXA9 cells vs hTERT/E6/E7-MSCV control cells. The legend shows the z-scores (red and green indicate over- and under-expressed genes, respectively). Rows correspond to genes, and columns correspond to replicates. Only genes with an adjusted $p < 0.05$ are shown, and arranged based on their fold-change.

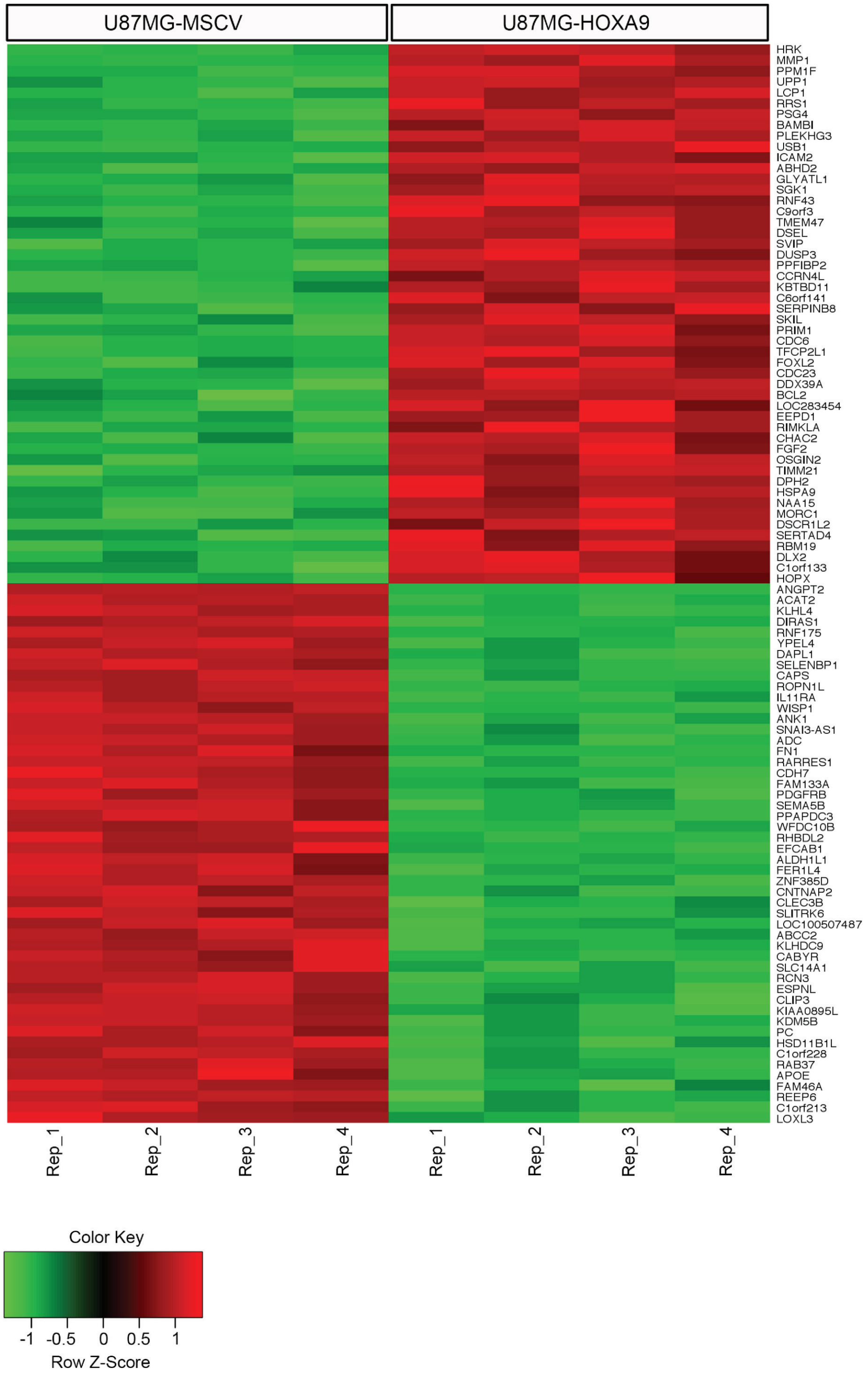

Supplementary Figure 5: Heatmap representation of the top 50 most up-regulated and top 50 most down-regulated genes in U87MG-HOXA9 cells vs U87MG-MSCV control cells. The legend shows the z-scores (red and green indicate over- and under-expressed genes, respectively). Rows correspond to genes, and columns correspond to replicates. Only genes with an adjusted $p < 0.05$ are shown, and arranged based on their fold-change.

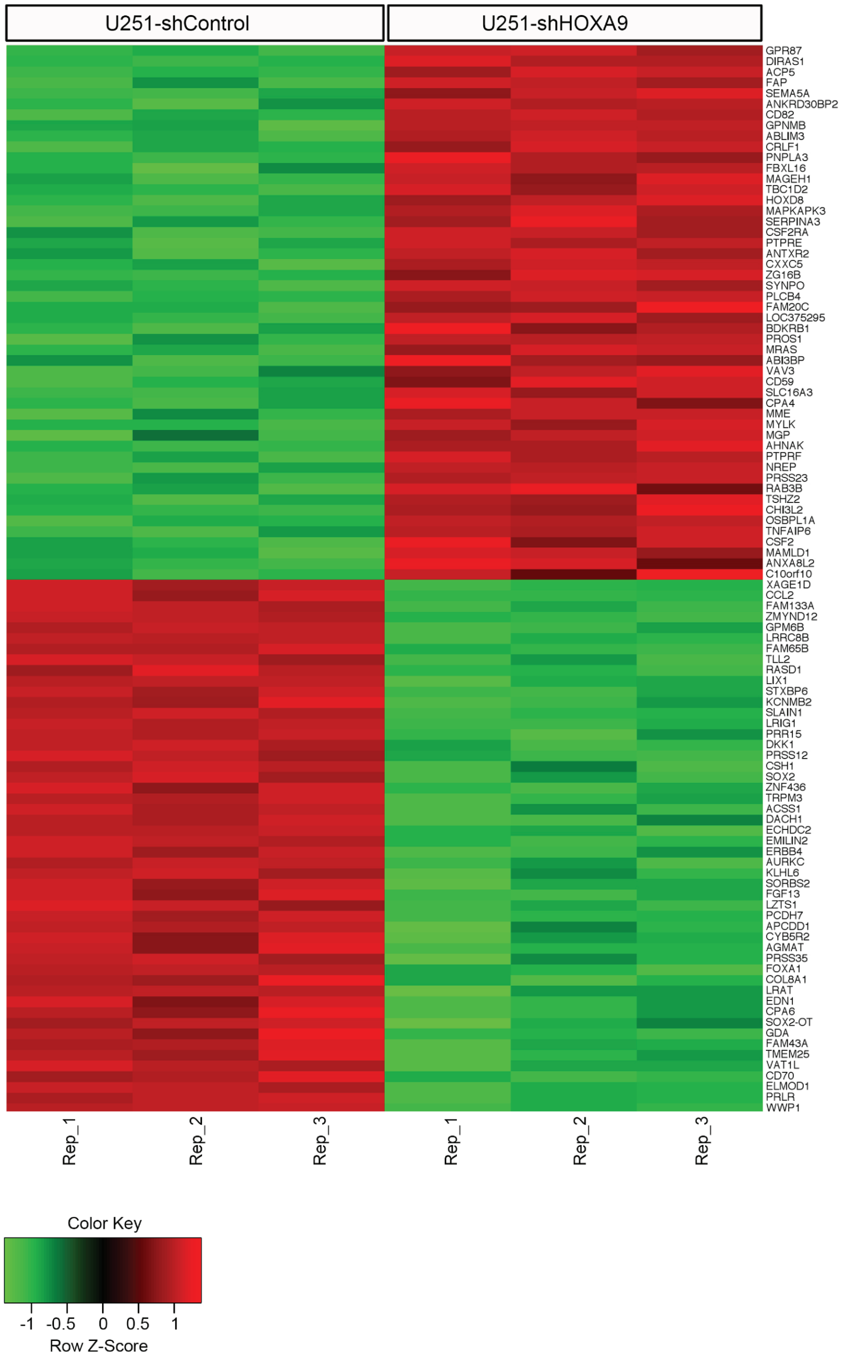

Supplementary Figure 6: Heatmap representation of the top 50 most up-regulated and top 50 most down-regulated genes in U251-shControl cells vs U251-shHOXA9 cells. The legend shows the z-scores (red and green indicate over- and under-expressed genes, respectively). Rows correspond to genes, and columns correspond to replicates. Only genes with an adjusted $p < 0.05$ are shown, and arranged based on their fold-change.

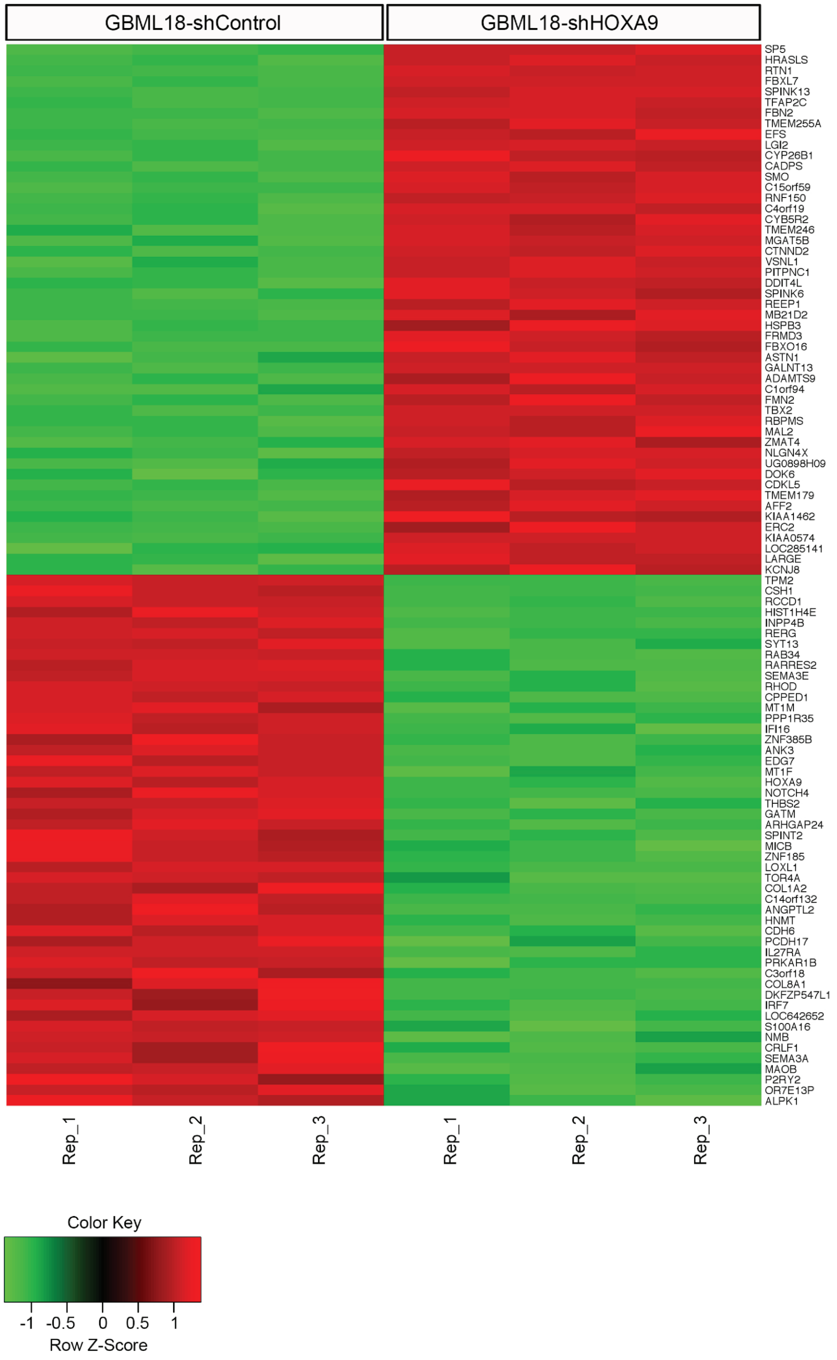

Supplementary Figure 7: Heatmap representation of the top 50 most up-regulated and top 50 most down-regulated genes in GBML18-shControl cells vs GBML18-shHOXA9 cells. The legend shows the z-scores (red and green indicate over- and under-expressed genes, respectively). Rows correspond to genes, and columns correspond to replicates. Only genes with an adjusted $p < 0.05$ are shown, and arranged based on their fold-change.

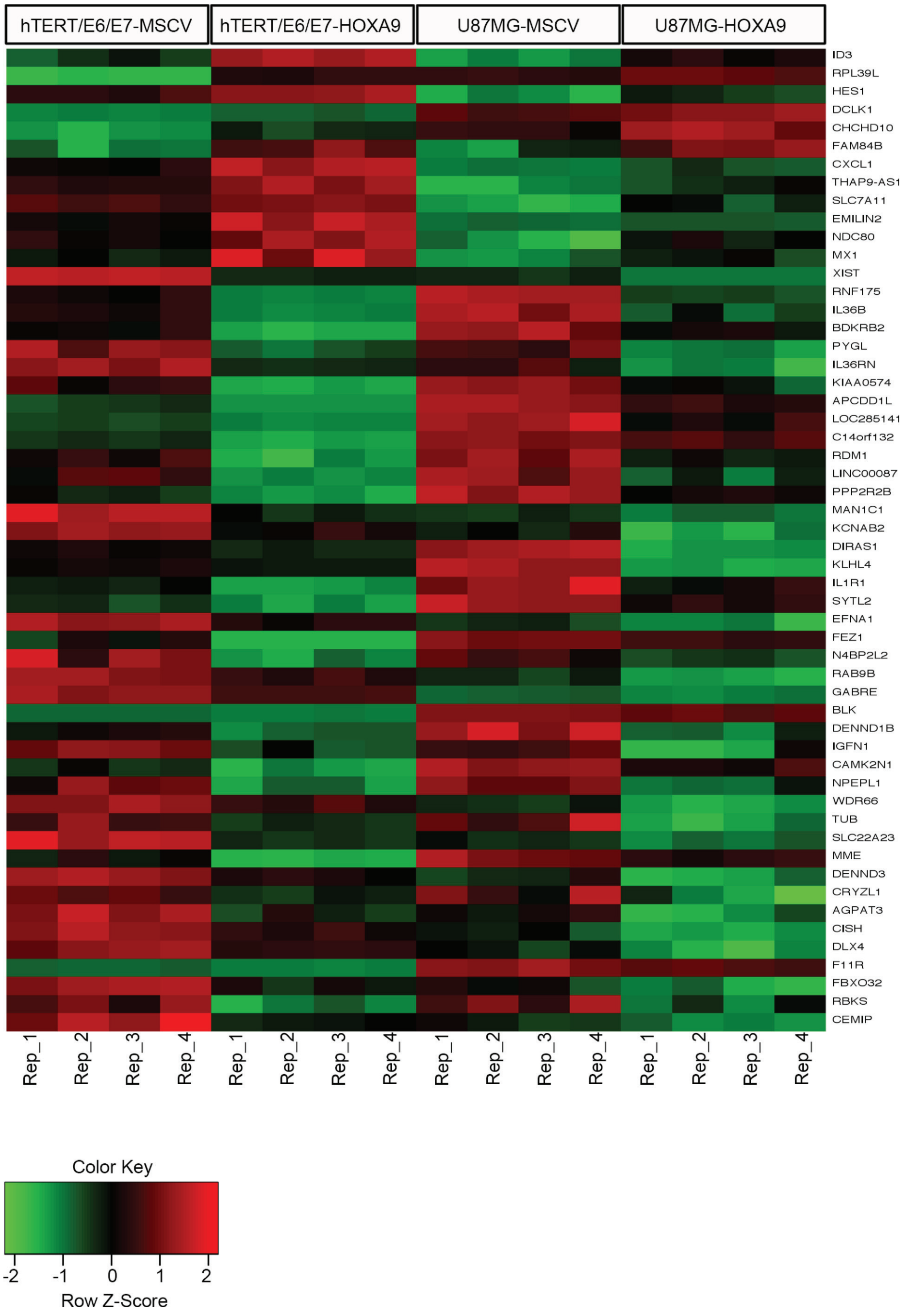

Supplementary Figure 8: Heatmap representation of the genes concomitantly differentially expressed in U87MG-HOXA9 and hTERT/E6/E7-HOXA9 cells vs their respective MSCV control cells. The legend shows the z-scores (red and green indicate over- and under-expressed genes, respectively). Rows correspond to genes, and columns correspond to replicates. Only genes with an adjusted $p < 0.05$ are shown, and arranged based on their fold-change.

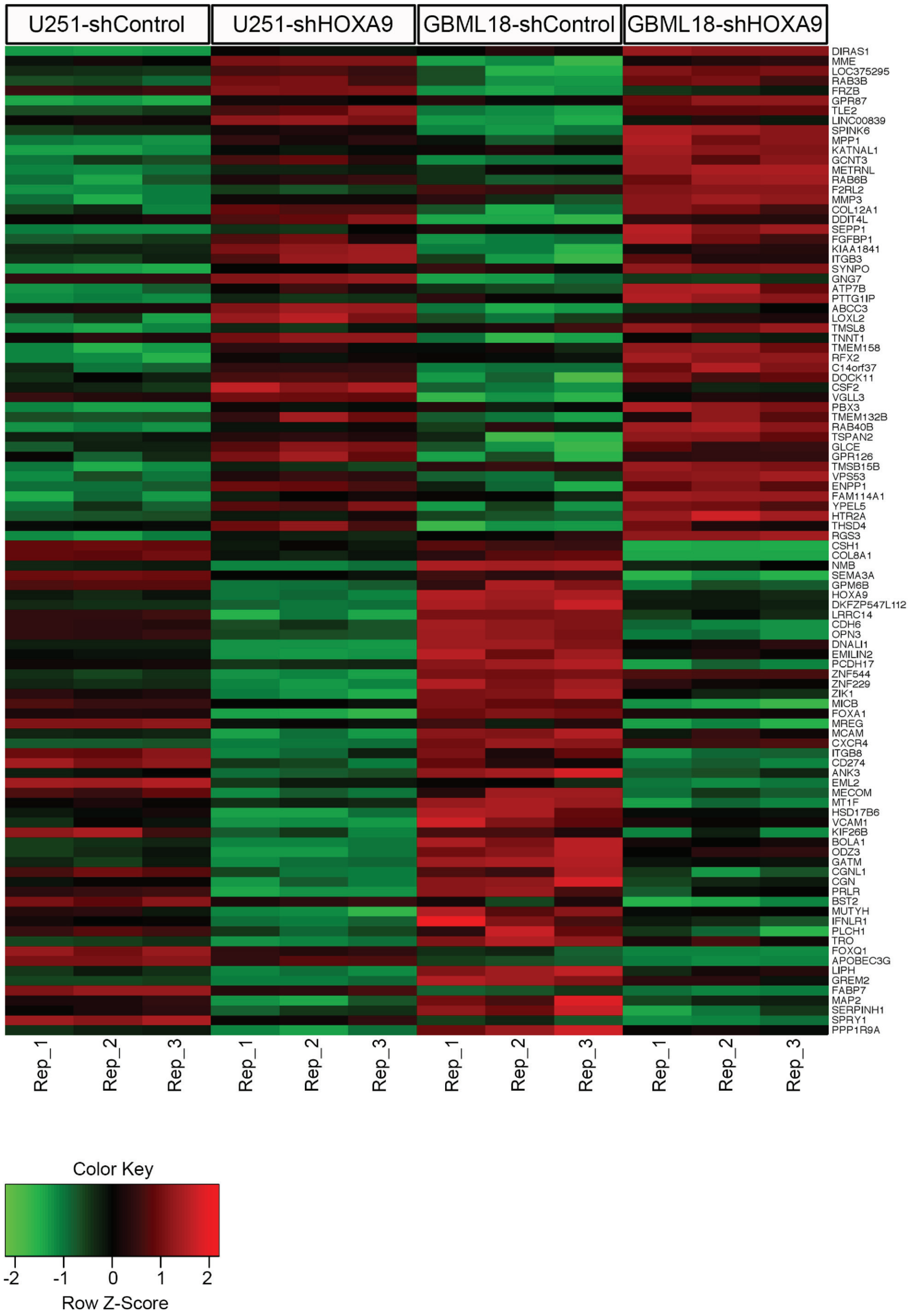

Supplementary Figure 9: Heatmap representation of the genes concomitantly differentially expressed in U251-shHOXA9 and GBML18-shHOXA9 cells vs their respective shControl cells. The legend shows the z-scores (red and green indicate over- and under-expressed genes, respectively). Rows correspond to genes, and columns correspond to replicates. Only genes with an adjusted $p < 0.05$ are shown, and arranged based on their fold-change.

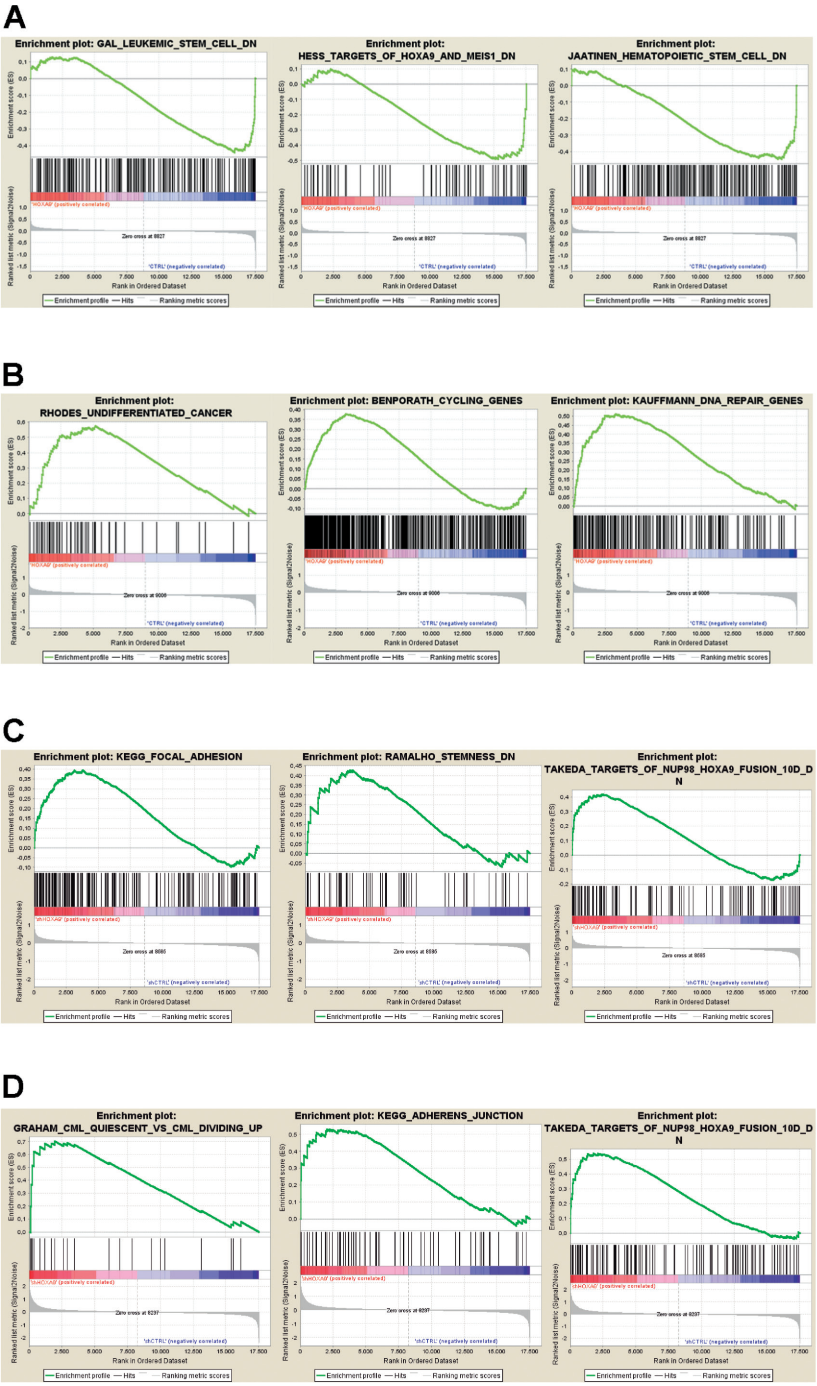

Supplementary Figure 10: HOXA9 transcriptome is enriched in several gene expression datasets related with critical cancer hallmark features. **(A)** Gene set enrichment analysis (GSEA) reveals that the HOXA9 transcriptome in hTERT/E6/E7 cell line is inversely associated with gene expression signatures of stem-like cells. **(B)** In U87MG cells, the HOXA9 transcriptome is enriched for transcriptomic signatures associated with cell cycling, undifferentiated cancer cells and DNA repair. **(C)** The transcriptome of HOXA9-silenced U251 cells is associated with genes depleted in embryonic, neural and hematopoietic stem cells, and with genes associated with cell-matrix adhesion. **(D)** The transcriptome of HOXA9-silenced GBML18 cells is associated with genes up-regulated in quiescent chronic myeloid leukemia and cell-cell adherens junctions.

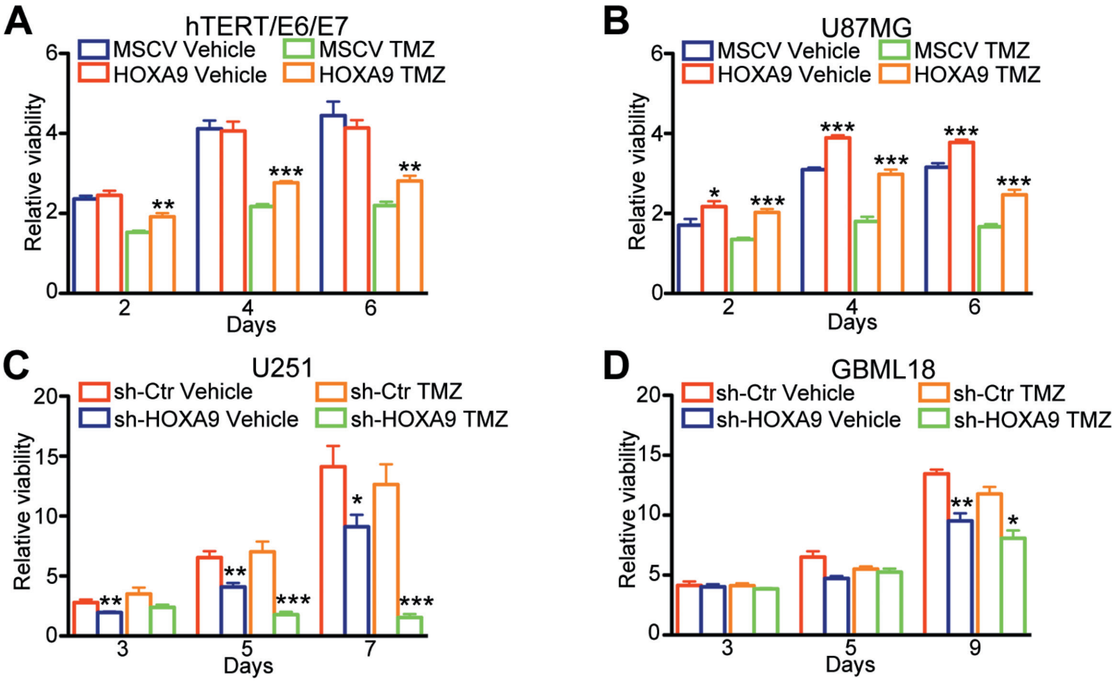

Supplementary Figure 11: Functional roles of *HOXA9* in GBM cell viability. **(A–D)** Metabolic cell viability MTS assays in *HOXA9*-negative/low or *HOXA9*–positive/high hTERT/E6/E7 (A), U87MG (B), U251 (C) and GBML18 (D) cells, between 2 and 9 days of treatment with TMZ or vehicle. Results are representative of three independent experiments (data points represent mean ± standard deviation). Statistical differences were calculated by Student *t*-tests (*$p < 0.05$; **$p < 0.01$; ***$p < 0.001$).

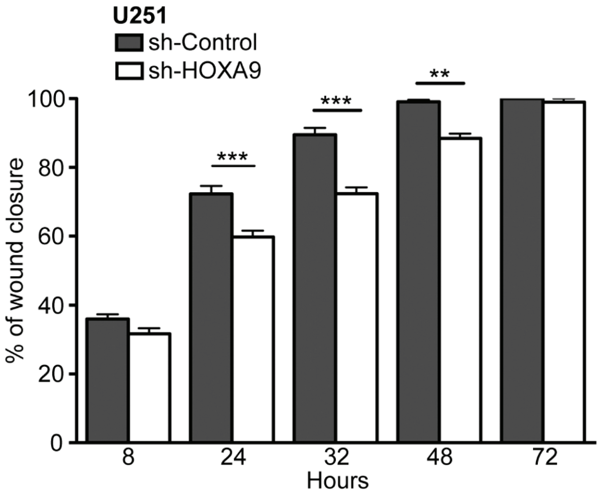

Supplementary Figure 12: *HOXA9* increases the migration capacity of U251 cells. The wound healing capacity of U251 cells was evaluated from 8 to 72 h after wound formation. U251-shControl cells migrate faster than U251-shHOXA9 cells, indicating *HOXA9* affects the migration dynamics of these cells. Results are representative of three independent experiments (data represented as mean ± standard deviations). Statistical differences were calculated by Student *t*-tests (**$p < 0.01$; ***$p < 0.001$).

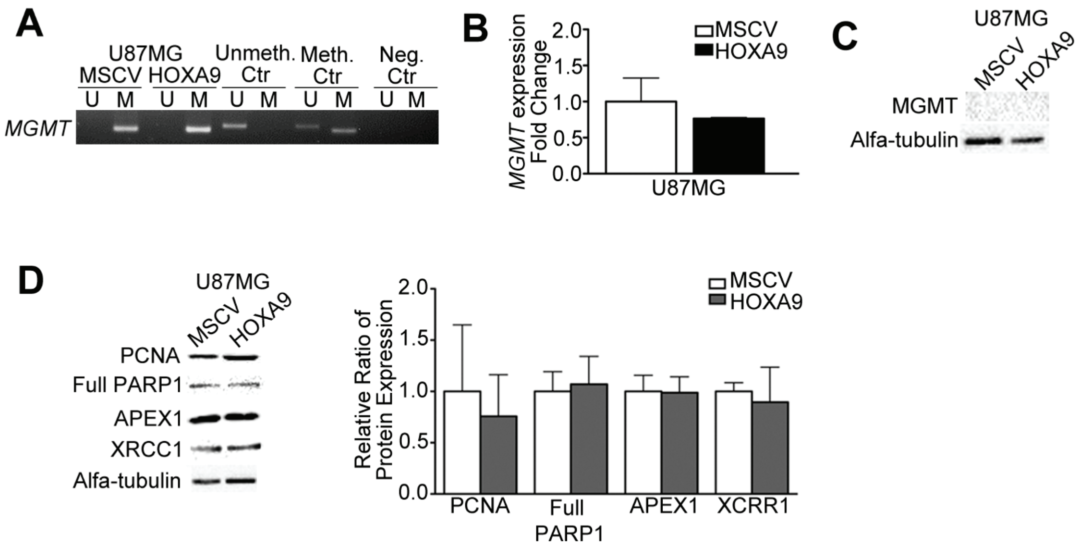

Supplementary Figure 13: *HOXA9* mediates response to temozolomide independently of MGMT and base excision repair (BER) family proteins. (A) *MGMT* promoter methylation analysis by methylation-specific PCR. **(B)** qPCR analysis for *MGMT* mRNA expression in U87MG *HOXA9*-positive and *HOXA9*-negative cells. **(C)** Western blots to MGMT and base excision repair (BER) family proteins **(D)**, showing no significant differences between U87MG-MSCV and U87MG-HOXA9 cells.

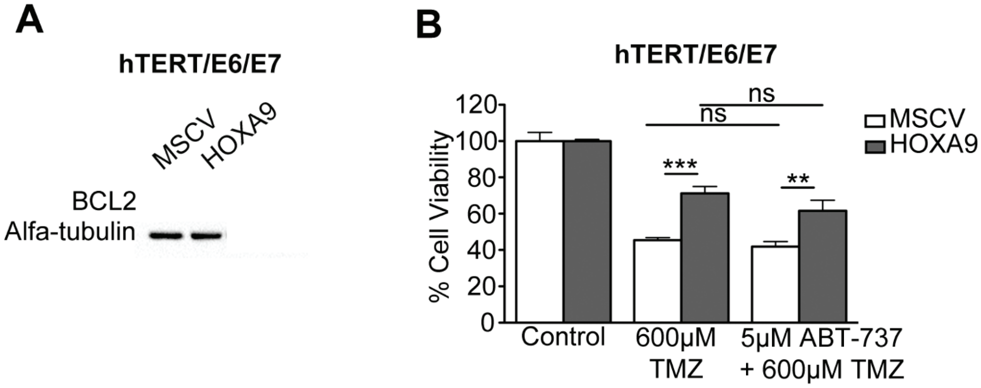

Supplementary Figure 14: Evaluation of specificity of BCL2 inhibitor ABT-737. (A) BCL2 protein levels were undetectable in hTERT/E6/E7 human immortalized astrocytes, regardless of *HOXA9* expression. **(B)** Concordantly, addition of ABT-737 to cells exposed to temozolomide (TMZ) did not affect cell viability as compared to TMZ alone (in contrast to BCL2-positive cells as shown in Figure 6). Results are representative of three independent experiments, performed in triplicates (data points represent mean ± standard deviation; **$p < 0.01$; ***$p < 0.001$; ns - not significant).

Supplementary Table 1: Selection of cancer-related gene sets obtained by gene set enrichment analysis (GSEA) of the HOXA9-transcriptome in HTERT/E6/E7, U87MG, U251 and GBML18 cells

	Gene Set Standard Name	Size	ES	FDR q-val
hTERT/E6/E7	Alonso_metastasis_DN	21	−0.70	0.137
	Takeda_targets_of_NUP98_HOXA9_fusion_6hr_DN	32	−0.58	0.184
	Huang_Dasatinib_Resistance_DN	56	−0.57	0.137
	Winnepenninckx_Melanoma_metastasis_DN	41	−0.55	0.213
	Beier_Glioma_Stem_Cell_DN	51	−0.54	0.192
	Hess_Targets_of_HOXA9_and_MEIS1_DN	63	−0.49	0.240
	Poola_Invasive_Breast_Cancer_DN	119	−0.49	0.182
	Schlesinger_Methylated_de_novo_in_cancer	80	−0.48	0.221
	Riggins_Tamoxifen_Resistance_DN	181	−0.47	0.169
	Vantveer_Breast_Cancer_Metastasis_DN	89	−0.46	0.247
	Jaatinen_Hematopoietic_Stem_Cell_DN	183	−0.45	0.204
	Gal_Leukemic_Stem_Cell_DN	196	−0.44	0.207
U87MG	Poola_Invasive_Breast_Cancer_Dn	119	−0.46	0.037
	Beier_Glioma_Stem_Cell_Dn	51	−0.46	0.124
	Ramalho_Stemness_Dn	58	−0.44	0.144
	Rhodes_Undifferentiated_Cancer	58	0.58	< 0.0001
	Benporath_Proliferation	121	0.60	< 0.0001
	Benporath_Cycling_genes	499	0.38	0.007
	Chang_Cycling_genes	34	0.71	< 0.0001
	Reactome_Transcription	177	0.50	< 0.0001
	Kauffmann_DNA_Repair_genes	195	0.51	< 0.0001
	Reactome_Synthesis_of_DNA	82	0.59	< 0.0001
	Winnepenninckx_Melanoma_metastasis_UP	121	0.52	< 0.0001
	Kang_Doxorubicin_resistance_UP	45	0.72	< 0.0001
	Hess_Targets_of_HOXA9_and_MEIS1_UP	56	0.51	0.006
U251	Takeda_Targets_Of_Nup98_Hoxa9_Fusion_10d_Up	143	−0.56	0.001
	Reactome_Cell_Cycle_Checkpoints	101	−0.45	0.161
	Reactome_Regulation_Of_Mitotic_Cell_Cycle	74	−0.48	0.170
	Hess_Targets_Of_Hoxa9_And_Meis1_Up	58	−0.48	0.221
	Dorsam_Hoxa9_Targets_Up	30	−0.54	0.222
	Beier_Glioma_Stem_Cell_Dn	52	0.50	0.112
	Colin_Pilocytic_Astrocytoma_Vs_Glioblastoma_Up	32	0.54	0.138
	Takeda_Targets_Of_Nup98_Hoxa9_Fusion_10d_Dn	121	0.42	0.144
	Kegg_Focal_Adhesion	179	0.39	0.141
	Kegg_Gap_Junction	68	0.46	0.131
	Ramaswamy_Metastasis_Dn	54	0.48	0.136
	Ramalho_Stemness_Dn	62	0.43	0.225
	Rizki_Tumor_Invasiveness_2d_Dn	54	0.42	0.250

(*Continued*)

	Gene Set Standard Name	Size	ES	FDR q-val
GBML18	Hummerich_Skin_Cancer_Progression_Up	80	−0.56	0.051
	Reactome_Rna_Pol_I_Rna_Pol_Iii_And_Mitochondrial_Transcription	101	−0.53	0.035
	Reactome_Integrin_Cell_Surface_Interactions	77	−0.49	0.191
	Reactome_Extracellular_Matrix_Organization	82	−0.47	0.187
	Lu_Tumor_Vasculature_Up	22	−0.63	0.201
	Reactome_Telomere_Maintenance	61	−0.55	0.090
	Nakamura_Cancer_Microenvironment_Up	20	−0.67	0.163
	Reactome_Meiosis	91	−0.48	0.193
	Reactome_Rna_Pol_I_Promoter_Opening	49	−0.65	0.022
	Takeda_Targets_Of_Nup98_Hoxa9_Fusion_10d_Dn	121	0.54	0.161
	Graham_Cml_Quiescent_Vs_Cml_Dividing_Up	22	0.70	0.213
	Odonnell_Metastasis_Dn	20	0.74	0.144
	Le_Neuronal_Differentiation_Up	15	0.75	0.210

ES: Enrichment Score; **FDR q-val:** False Discovery Rate *q*-value.

Supplementary Table 2: List of antibodies used for immunofluorescence, immunohistochemistry, and Western blot

Antibody	Dilution IF	Dilution IHC	Dilution WB	Source	# catalog
Nestin	1:100	1:100	-	Millipore	MAB5326
Alexa Fluor® 488 Goat Anti-Mouse IgG (H+L)	1:1000	-	-	Invitrogen	A-11001
Cyclin D1	-	1:100	-	Cell Signaling	2978S
BCL2	-	1:200	-	Cell Signaling	2870S
MSH6	-	1:500	1:500	BD Biosciences	610919
Ki-67	-	1:200	-	BD Biosciences	550609
CD31	-	1:200	-	Santa Cruz Biotechnology	sc-1506
Biotinylated horse anti-goat	-	1:500	-	Vector Labs	BA9500
MGMT	-	-	1:500	Zymed	357000
MLH1	-	-	1:500	Pharmingen	554073
MLH3	-	-	1:500	Santa Cruz Biotechnology	sc-25313
PCNA	-	-	1:500	Santa Cruz Biotechnology	sc-25280
α-tubulin	-	-	1:200	Santa Cruz Biotechnology	sc-23948
MSH2	-	-	1:500	Calbiochem	NA27
MSH3	-	-	1:250	BD Biosciences	611390
PMS2	-	-	1:500	BD Biosciences	556415
PARP1/2	-	-	1:1000	Cell Signaling	9532S
XRCC1	-	-	1:500	Cell Signaling	2735S
APE1	-	-	1:500	Novus Biochemicals	NB100–116
HRP conjugated anti-mouse IgG	-	-	1:1000	Santa Cruz Biotechnology	sc-2031
HRP conjugated anti-rabbit IgG	-	-	1:1000	Santa Cruz Biotechnology	sc-2004

H + L: Heavy and Light immunoglobulin G chains; **HRP:** Horseradish peroxidase; **IF:** Immunofluorescence; **IHC:** Immunohistochemistry; **WB:** Western blot.

Supplementary Table 3: Sequence of primers used for PCR analyses

Gene	Primer Sense	Primer Antisense	Primer Tm (°C)
ICAM2	GGATCCCAGAGCTACCCTTC	CGTGTCATGGGAGATGTTTG	59
NPR3	GTGGCCTAGAAGAATCGGCA	GGGTTCGCCTCTCAATGGTT	60
BAMBI	CTTGCAAGCACGACAGACAT	GAAGTCAGCTCCTGCACCTT	58
C10orf10	GACTCAGTGCACTGTCCTCC	GACCTCATCACTCTGGCGAG	60
ANGPT2	ATAAGCAGCATCAGCCAACC	CCTTGAGCGAATAGCCTGAG	57
PDGFRB	ATAAGCAGCATCAGCCAACC	CCTTGAGCGAATAGCCTGAG	61
RAC2	CAGCACACCCATCATCCTG	CCTCTCTGGGTGAGAGCTGA	61
CXCL1	AGGGAATTCACCCCAAGAAC	TGTTCAGCATCTTTTCGATGA	60
NDRG1	CTCGCTGAGGCCTTCAAGTA	AGAGAAGTGACGCTGGAACC	60
DKK1	CAGGCGTGCAAATCTGTCT	CCCATCCAAGGTGCTATGAT	60
SOX2	CCCACCTACAGCATGTCCTA	CTGATCATGTCCCGGAGGT	60
TOX2	CTTCCCGCACATCTCTGAGT	TGAGGTAGAGCGATTTGTCC	58
HOXA9	GCCCGTGCAGCTTCCAGTCC	GAGCGCGCATGAAGCCAGTTG	61
GUSB	CCTGTGACCTTTGTGAGCAA	GTGCCCGTAGTCGTGATACC	57
TBP	GAGCTGTGATGTGAAGTTTCC	TCTGGGTTTGATCATTCTGTAG	60
***HRPT1* (human)**	TGAGGATTTGGAAAGGGTGT	GAGCACACAGAGGGCTACAA	60
ACTIN, beta	GGACTTCGAGCAAGAGATGG	AGCACTGTGTTGGCGTACAG	59
MGMT	GCCGGCTCTTCACCATCCCG	GCTGCAGACCACTCTGTGGCACG	60
***HPRT1* (mouse)**	GCTGGTGAAAAGGACCTCT	CACAGGACTAGAACACCTGC	58
BCL2	ATGTGTGTGGAGAGCGTCAA	TTCAGAGACAGCCAGGAGAAA	61–56 (−1ºC/cycle)

Tm: Melting temperature.

For all genes, PCR parameters were as follows: 4 minutes at 94ºC, 35 cycles of denaturation for 30 seconds at 94ºC, annealing for 30 seconds (at specific primer Tm temperature), extension at 72ºC for 30 seconds, and final extension at 72ºC for 8 minutes. For qPCR, all parameters were identical to conventional PCR, except the number of cycles that was extended to 45, and a final extension was performed by increasing the temperature in 1ºC each 5 seconds from 65ºC to 95ºC.
